# Major Contribution of Somatostatin-Expressing Interneurons and Cannabinoid Receptors to Increased GABA Synaptic Activity in the Striatum of Huntington’s Disease Mice

**DOI:** 10.3389/fnsyn.2019.00014

**Published:** 2019-05-14

**Authors:** Sandra M. Holley, Laurie Galvan, Talia Kamdjou, Ashley Dong, Michael S. Levine, Carlos Cepeda

**Affiliations:** Intellectual and Developmental Disabilities Research Center, Department of Psychiatry and Biobehavioral Sciences, Jane and Terry Semel Institute for Neuroscience and Human Behavior, Brain Research Institute, University of California, Los Angeles, Los Angeles, CA, United States

**Keywords:** striatum, Huntington’s disease (HD), GABA interneurons, CB1 receptors, electrophysiology, optogenetics

## Abstract

Huntington’s disease (HD) is a heritable neurological disorder that affects cognitive and motor performance in patients carrying the mutated *huntingtin (HTT)* gene. In mouse models of HD, previous reports showed a significant increase in spontaneous GABA_A_ receptor-mediated synaptic activity in striatal spiny projection neurons (SPNs). In this study, using optogenetics and slice electrophysiology, we examined the contribution of γ-aminobutyric acid (GABA)-ergic parvalbumin (PV)- and somatostatin (SOM)-expressing interneurons to the increase in GABA neurotransmission using the Q175 (heterozygote) mouse model of HD. Patch clamp recordings in voltage-clamp mode were performed on SPNs from brain slices of presymptomatic (2 months) and symptomatic (8 and 12 months) Q175 mice and wildtype (WT) littermates. While inhibitory postsynaptic currents (IPSCs) evoked in SPNs following optical activation of PV- and SOM-expressing interneurons differed in amplitude, no genotype-dependent differences were observed at all ages from both interneuron types; however, responses evoked by either type were found to have faster kinetics in symptomatic mice. Since SOM-expressing interneurons are constitutively active in striatal brain slices, we then examined the effects of acutely silencing these neurons in symptomatic mice with enhanced *Natronomonas pharaonis* halorhodopsin (eNpHR). Optically silencing SOM-expressing interneurons resulted in a greater decrease in the frequency of spontaneous IPSCs (sIPSCs) in a subset of SPNs from Q175 mice compared to WTs, suggesting that SOM-expressing interneurons are the main contributors to the overall increased GABA synaptic activity in HD SPNs. Additionally, the effects of activating GABA_B_ and cannabinoid (CB1) receptors were investigated to determine whether these receptors were involved in modulating interneuron-specific GABA synaptic transmission and if this modulation differed in HD mice. When selectively activating PV- and SOM-expressing interneurons in the presence of the CB1 receptor agonist WIN-55,212, the magnitudes of the evoked IPSCs in SPNs decreased for both interneuron types although this change was less prominent in symptomatic Q175 SPNs during SOM-expressing interneuron activation. Overall, these findings show that dysfunction of SOM-expressing interneurons contributes to the increased GABA synaptic activity found in HD mouse models and that dysregulation of the endocannabinoid system may contribute to this effect.

## Introduction

Huntington’s disease (HD) is a fatal autosomal dominant genetic disorder caused by an unstable expansion of cytosine–adenine–guanine (CAG) repeats in exon 1 of the *huntingtin (HTT)* gene (MacDonald et al., 1993). Individuals affected with HD exhibit symptoms such as loss of motor coordination, cognitive impairment and psychiatric disturbances that progress in severity with age (Walker, 2007; Bates et al., 2015; Snowden, 2017). The main neuropathological feature of HD is atrophy of the striatum, as well as the cerebral cortex, hippocampus, thalamus, hypothalamus and cerebellum (Waldvogel et al., 2015). In the striatum, there is massive degeneration and loss of spiny projection neurons (SPNs), the main cell type involved in relaying integrated information from the cortex and thalamus to output structures of the basal ganglia (Albin et al., 1990).

SPNs are γ-aminobutyric acid (GABA)-ergic cells that represent over 90% of all striatal neurons and form labyrinthine connections with other SPNs and striatal interneurons (reviewed by Bolam et al., 2000; Chuhma et al., 2011). The remaining cell types in the striatum are interneurons that modulate intra-striatal communication in addition to SPN output. While SPN degeneration and loss are abundant in early and late stages of HD, other striatal cells also are affected. In symptomatic HD patients and mouse models, the number of GABAergic parvalbumin (PV)-expressing interneurons is decreased and their dendritic arborization is greatly diminished (Reiner et al., 2013; Simmons et al., 2013; Paldino et al., 2017; Holley et al., 2019). Furthermore, while large cholinergic and GABAergic somatostatin (SOM)-expressing interneurons appear to be spared in HD, both types of interneurons display altered physiology in symptomatic HD mice (Holley et al., 2015, 2019; Tanimura et al., 2016). These disease-related dysfunctions in individual cell populations add stress on striatal microcircuits leading to altered striatal output generally associated with abnormal movements.

GABA-releasing interneurons make up about 5% of all striatal cells and one of their primary roles is to modulate SPN output. Recent reports show that the diversity of striatal GABAergic interneurons is greater than previously thought (reviewed by Tepper et al., 2018). There are three main types, low-threshold spiking (LTS) interneurons, which are SOM-, neuropeptide Y (NPY)-, and nitric oxide synthase (NOS)-expressing, fast-spiking (FS) PV-expressing interneurons and calretinin-expressing interneurons. All but the latter have been characterized in brains of healthy and HD mice (Tepper et al., 2010; Cepeda et al., 2013; Holley et al., 2019). Fast-spiking interneurons (FSIs) display fast-firing properties and mediate feed-forward inhibition, while the LTS interneurons fire spontaneously at lower frequencies and, in addition to GABA, they release neuromodulators that may have neuroprotective attributes (Kumar, 2008; Rajput et al., 2011).

There is growing evidence that GABA neurotransmission is abnormal in animal models of HD (Cepeda et al., 2004, 2010; Centonze et al., 2005; Andre et al., 2011; Dvorzhak et al., 2013; Indersmitten et al., 2015; Hsu et al., 2018). Further studies from our laboratory demonstrated alterations in intrinsic and synaptic properties in both FSIs and LTS interneurons which may contribute to the increased striatal GABA transmission (Cepeda et al., 2013; Holley et al., 2019). While there are multiple sources of GABA inhibition in the striatum of HD model mice, the underlying mechanisms are not well understood. In an attempt to elucidate the key cell type(s) involved in increased inhibition we used optogenetics to selectively manipulate (activate or inhibit) PV- or SOM-expressing interneurons in the Q175 mouse model of HD to determine how evoked responses and spontaneous GABA synaptic activity in SPNs are altered throughout disease progression. In addition, we investigated whether activation of GABA_B_ and endocannabinoid-dependent signaling affects interneuron-specific inhibitory synaptic transmission in SPNs and whether these signaling pathways are altered in HD mice.

## Materials and Methods

### Mice

Experimental procedures were performed in accordance with the United States Public Health Service Guide for Care and Use of Laboratory Animals and were approved by the Institutional Animal Care and Use Committee at the University of California Los Angeles (UCLA). All mice were obtained from our breeding colonies and every effort was made to minimize pain, discomfort, and the number of mice used. Animal housing conditions were maintained under a standard 12 h light/dark cycle (light cycle starting at 6 AM and ending at 6 PM) and at a temperature of 20–26°C. The animals had *ad libitum* access to food and water. Total numbers of mice used for all experiments were 105 wildtype (WT; 63 males, 42 females) and 111 heterozygous Q175 mice (50 males, 61 females). In order to limit the number of mice used, wherever feasible, multiple experiments were performed using brain slices from the same mouse. In all experiments mice were examined at presymptomatic (2–3 month; mean age 81 ± 2 and 80 ± 2 days for WT and Q175, respectively) and fully symptomatic (10.5–15 month; mean age 408 ± 5 and 413 ± 5 days for WT and Q175, respectively) stages. In selected experiments, a third group was added (8–9 month; mean age 265 ± 3 days for both WT and Q175). Mice from this latter group are also symptomatic but the phenotype is not as severe as in older mice (Heikkinen et al., 2012). For optogenetic experiments involving PV- and SOM-expressing interneurons, heterozygous Q175 mice (CHDI, Inc.) were crossed with homozygous PV- or SOM-Cre mice (B6.129P2-Pvalb^tm1(cre)Arbr^/J, RRID:IMSR_JAX:008069 and Sst^tm2.1(cre)Zjh^/J, RRID:IMSR_JAX:013044, The Jackson Laboratory) to produce heterozygote Q175 mice with Cre expression in PV- or SOM-expressing interneurons. To examine differential inputs to direct and indirect pathway neurons, Drd1a-tdTomato mice [B6.Cg-Tg(Drd1a-tdTomato)6Calak/J, RRID:IMSR_JAX:016204, The Jackson Laboratory] were crossed with heterozygous Q175 mice to produce mice with D1 cells labeled with tdTomato. Additionally, 17 R6/2 mice (11 males, six females) and 14 WT littermates (six males, eight females) from our colony were used in selected experiments. For these mice, WT male C57BL/6xCBA mice were crossed with WT female C57BL/6xCBA mice that had transplanted R6/2 ovaries [B6CBA-Tg(HDexon1)62Gpb/3J, RRID:IMSR_JAX:006494, The Jackson Laboratory]. For experiments investigating alterations in SPN responses induced by activation of dopamine receptor-expressing (D1)-SPNs, we crossed WT and Q175 mice with D1-Cre mice [B6.FVB(Cg)-Tg(Drd1-cre)EY262Gsat/Mmucd, RRID:MMRRC_030989-UCD]. Genotyping was performed using PCR of DNA obtained from tail samples, once at weaning and again following use to confirm the genotype. CAG repeat lengths were determined for breeders and for experimental animals by Laragen (Culver City, CA, USA). For Q175 mice, CAG repeats were 184.8 ± 1.4 (range 160–215) and for R6/2 mice, repeats were 158.1 ± 1.7 (range 144–165). Both male and female mice were used for all experiments. We observed no consistent differences between sexes and the data were pooled.

### Surgery

Animals were anesthetized with isoflurane and burr holes were drilled into the skull at the injection sites. For experiments involving the activation or silencing of striatal interneurons AAV2/1-EF1a-DIO-hChR2(H134R)-eYFP or AAV2/1-EF1a-DIO-eNpHR3.0-eYFP (University of Iowa Viral Vector Core Facility, Iowa City, IA or UNC Vector Core, Chapel Hill, NC, USA) was stereotactically injected bilaterally into the dorsolateral striata. The stereotaxic injection coordinates were (relative to Bregma): AP +1.0, ML ± 2.0, and DV −3.3 mm from the surface of the brain. For each AAV (titer 1.2–3.0 × 10^12^ vg/ml), a total of 1.0 μl (per hemisphere) was injected at a rate of 0.2 μl/min into WT and Q175 PV- or SOM-Cre mice. For selective activation of cortical (M1) inputs, mice were injected unilaterally at two sites (0.25 μl each) with AAV-CaMKII-hChR2(H134R)-eYFP (University of Iowa Viral Vector Core Facility, Iowa City, IA or UNC Vector Core, Chapel Hill, NC, USA) at a rate of 0.1 μl/min. Coordinates for cortical injections were (relative to Bregma): AP +1.7, ML ± 1.5, DV −1.2 and −0.5 mm from the surface of the brain. After each AAV injection (titer UNC 3.0–5.0 × 10^12^ vg/ml), the needle remained in place for 5–7 min before retraction in order to avoid vector backflow. Mice were sacrificed for electrophysiological and experiments ~4–6 weeks post-injection to ensure sufficient opsin expression.

### Brain Slice Electrophysiology

Mice were deeply anesthetized with isoflurane and perfused transcardially with an ice-cold, high sucrose-based solution containing (in mM): 26 NaHCO_3_, 1.25 NaH_2_PO_4_, 208 sucrose, 10 glucose, 2.5 KCl, 1.3 MgCl_2_, 8 MgSO_4_. Mice were decapitated, brains dissected out and immediately placed in the oxygenated slicing solution. Coronal or sagittal slices (300 μm) were cut and transferred to an incubating chamber containing artificial cerebrospinal fluid (ACSF; in mM): 130 NaCl, 3 KCl, 1.25 NaH_2_PO_4_, 26 NaHCO_3_, 2 MgCl_2_, 2 CaCl_2_, and 10 glucose oxygenated with 95% O_2_–5% CO_2_ (pH 7.2–7.4, osmolality 290–310 mOsm/L, 32–34°C). Slices were allowed to recover for an additional 30 min at room temperature. Brain slice recordings were limited to the dorsolateral region of the striatum and all recordings were performed at room temperature using upright microscopes (Olympus BX51WI or BX61WI) equipped with differential interference contrast optics and fluorescence imaging (QIACAM fast 1394 monochromatic camera with Q-Capture Pro software, QImaging). Whole-cell patch clamp recordings in voltage- and current-clamp modes were obtained using a MultiClamp 700B Amplifier (Molecular Devices) and the pCLAMP 10.3 or 10.5 acquisition software. The patch pipette (3–5 MΩ resistance) contained a cesium (Cs)-based internal solution (in mM): 125 Cs-methanesulfonate, 4 NaCl, 1 MgCl_2_, 5 MgATP, 9 EGTA, 8 HEPES, 1 GTP-Tris, 10 phosphocreatine, and 0.1 leupeptin (pH 7.2 with CsOH, 270–280 mOsm) for voltage-clamp recordings or a K-gluconate-based solution containing (in mM): 112.5 K-gluconate, 4 NaCl, 17.5 KCl, 0.5 CaCl_2_, 1 MgCl_2_, 5 ATP (K^+^ salt), 1 NaGTP, 5 EGTA, 10 HEPES, pH 7.2 (270–280 mOsm/L) for current-clamp recordings. All internal electrode solutions contained 0.2% biocytin for subsequent immunodetection and identification of recorded cells. In experiments where evoked excitatory postsynaptic currents (EPSCs) were recorded in SPNs following optical activation of cortical inputs, QX 314 Cl^−^ (4 mM) was included in the internal pipette solution to block activity-dependent Na^+^ channels. After breaking through the membrane, cell properties (capacitance, input resistance, decay time constant) were recorded in voltage-clamp mode while holding the membrane potential at −70 mV. Resting membrane potentials were recorded in current-clamp mode. Data were omitted if electrode access resistance exceeded 30 MΩ.

For optogenetic experiments, PV- or SOM-expressing interneurons or D1 receptor-expressing SPNs were activated with a single light pulse (470 nm, 0.5 ms, 3 mW, CoolLED) delivered through the epifluorescence illumination pathway using Chroma Technologies filter cubes. Evoked inhibitory postsynaptic currents (IPSCs) in response to optical activation were recorded in voltage-clamp mode, at a holding potential of +10 mV and in the presence of 2,3-Dioxo-6-nitro-1,2,3,4-tetrahydrobenzo[f]quinoxaline-7-sulfonamide (NBQX, 10 μM) and DL-2-Amino-5-phosphonopentanoic acid (APV, 50 μM) to block α-amino-3-hydroxy-5-methyl-4-isoxazolepropionic acid (AMPA) and N-methyl-D-aspartate (NMDA) receptors, respectively. In some cells, 10 μM bicuculline (BIC) was applied to block optically-evoked IPSC responses, confirming they were due to activation of GABA_A_ receptors. In our recording conditions, based on the Cl^−^ concentration in the internal solution and the addition of NBQX/APV in the bath, the E_Cl_ was −55.0 ± 1.6 mV (*n* = 8). For stimulation of channelrhodopsin (ChR2)-expressing corticostriatal projections in decorticated coronal slices, a single light pulse (470 nm, 1 ms, 5 mW) was delivered and evoked EPSCs were recorded in SPNs while holding the membrane at −70 mV and in the presence of BIC (10 μM). For experiments where SOM-expressing interneurons were silenced with optical activation of enhanced *Natronomonas pharaonis* halorhodopsin (eNpHR), yellow light (585 nm, 1 mW) was applied at various durations through the microscope objective. Recordings of spontaneous IPSCs (sIPSCs) were obtained from SPNs (60 s) prior to and (60 s) during yellow light illumination at a holding potential of +10 mV and in the presence of NBQX (10 μM) and APV (50 μM).

Following recordings, brain slices were fixed in 4% PFA for 24 h. Slices were then washed with 0.1 M PBS, permeabilized with 1% Triton overnight at 4°C, and incubated for 2 h with Alexa 594 conjugated streptavidin (1:1,000, ThermoFisher Scientific, Waltham, MA, USA) at room temperature. Fluorescent images from both recorded cells and eYFP-expressing terminals were obtained using a Zeiss confocal ApoTome equipped with 40× and 63× objectives.

The following drug reagents were obtained from Tocris Bioscience/Bio-Techne (Minneapolis, MN, USA): BIC, NBQX, APV, QX314 Cl^−^, the cannabinoid type 1 (CB1) receptor agonist WIN 55,212-2 and the antagonist, AM 251. All drug stocks were made using double-distilled H_2_O except for WIN 55,212-2 and AM 251 which were dissolved in DMSO. The final concentration of DMSO in recording bath solutions did not exceed 0.05%, a concentration that when tested in a few cells did not significantly alter cell membrane or evoked response properties following prolonged exposure (20 min).

### Analyses and Statistics

Analyses of optically-evoked postsynaptic responses were performed using the Clampfit 10.3 or 10.5 software (Molecular Devices). Evoked response peak amplitudes were calculated by measuring the difference between the highest absolute value point of the response and average baseline. Response areas were calculated using an integral function available in the Clampfit software that measures the area under the response curve relative to baseline. Decay times were calculated by measuring the time (in ms) the response took to decay from 90% to 10% of the peak amplitude (Parievsky et al., 2017). In addition, adjusted decay times and time constants were assessed by first normalizing the amplitudes of optically-evoked IPSCs or EPSCs amplitudes to the maximal WT response amplitude for each group (see Supplementary Material). For all optically-evoked responses, 3–6 sweeps were recorded and averaged (30 s inter-sweep interval). sIPSCs were analyzed off-line using the automatic threshold detection protocol within the Mini Analysis Program version 6.0 (Synaptosoft) and subsequently checked manually for accuracy. The threshold amplitude for the detection of an event (10 pA) was set 3× above the root mean square background noise level (max ~3 pA at *V*_hold_ = +10 mV). All statistical analyses were performed using SigmaPlot 13.0 software. Differences between group means were assessed with appropriate Student’s *t*-tests (unpaired) or Fisher exact tests for proportions, and appropriately designed analyses of variance (two-way ANOVAs, followed by Holm-Sidak or Bonferroni *post hoc* tests). Values in figures, tables and text are presented as mean ± SEM. Differences were considered statistically significant if *p* < 0.05 and are indicated with asterisks in the figures (*p < 0.05, **p < 0.01, ***p < 0.001, ****p < 0.0001).

## Results

### Expression and Activation of Channelrhodopsin in Striatal GABAergic Interneurons

**Figure 1:**
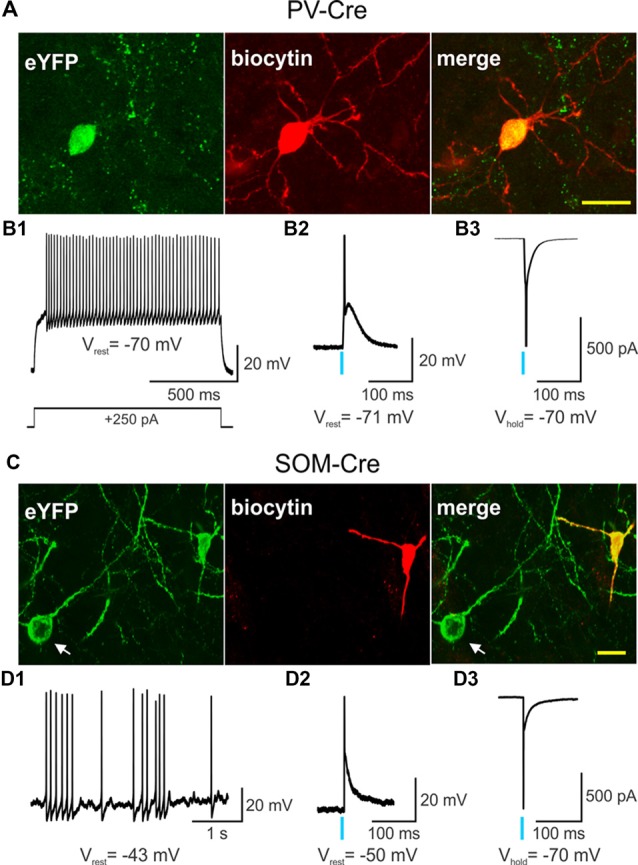
Single action potentials are generated by a brief light pulse in parvalbumin (PV)- and somatostatin (SOM)-expressing interneurons. Confocal images of biocytin-filled PV- **(A)** or SOM-expressing **(C)** interneurons (red) that also express eYFP from 2 months-old wildtype (WT) PV- and SOM-Cre mice. In **(C)** the cell body of a SOM-expressing interneuron that also expresses eYFP but was not recorded from is visible (white arrow). Scale bar = 20 μm. **(B1)** Voltage response to a depolarizing, suprathreshold current injection in a PV-expressing interneuron that also expresses channelrhodopsin-2 (ChR2) and eYFP. Recordings of a PV-expressing interneuron co-expressing ChR2/eYFP in current-clamp **(B2)** and in voltage-clamp **(B3)** modes show that a brief light pulse (0.5 ms, 470 nm, 3 mW) produces an action potential. The action potential in the bottom trace was truncated for the figure. **(D1)** Spontaneous firing of a SOM-expressing interneuron that expresses ChR2/eYFP in current-clamp mode. A brief light pulse (0.5 ms, 470 nm, 3 mW) produces an action potential in a SOM interneuron recorded in current-clamp **(D2)** and in voltage-clamp **(D3)** modes. The action potential in the bottom trace was truncated for the figure. In this and in subsequent figures, a blue vertical line indicates the delivery point of a single blue light stimulation pulse.

ChR2 was selectively expressed in striatal PV- and SOM-expressing interneurons by stereotactically injecting a Cre-dependent AAV-ChR2-eYFP into WT and Q175 mice crossed with PV- and SOM-Cre mice, respectively (Figures 1A,C). Recordings from eYFP positive cells in PV-Cre (Figure 1B1) and SOM-Cre (Figure 1D1) mice showed distinct electrophysiological properties as previously reported (Tepper et al., 2010; Holley et al., 2019). Compared with SOM-expressing interneurons, PV-expressing interneurons were more hyperpolarized at rest, and displayed suprathreshold firing frequencies of 50–100 Hz. In contrast, SOM-expressing interneurons exhibited increased membrane input resistances, more depolarized resting membrane potentials, and fired spontaneously at frequencies between 1–10 Hz. After a brief pulse of blue light (0.5 ms, 470 nm), both PV- and SOM-expressing interneurons fired a single action potential as shown in current- (Figures 1B2,D2) and voltage-clamp recordings (Figures 1B3,D3). Thus, utilizing specific Cre-reporter mice combined with optogenetics, we were able to effectively activate striatal PV- or SOM-expressing interneurons in WT and Q175 mice.

### Responses of SPNs to Optogenetic Activation of PV- and SOM-Expressing Interneurons

**Figure 2:**
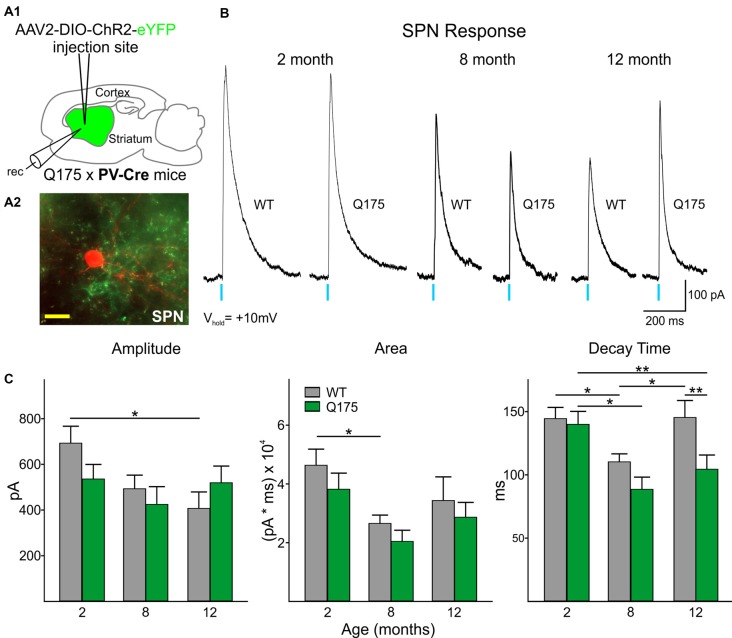
Optical activation of PV-expressing interneurons in Q175 and WT mice at three ages.** (A1)** Schematic of AAV injection site and recording area in Q175xPV-Cre mice. **(A2)** Confocal z-stack image of a biocytin-filled spiny projection neuron (SPN; red) that was recorded from a 15 months-old Q175 mouse. Processes expressing ChR2-eYFP can be seen surrounding the recorded cell. Scale bar = 20 μm. **(B)** Sample traces of evoked GABAergic responses in WT and Q175 SPNs from 2-, 8- and 12 months-old mice following optical activation of PV-expressing interneurons. **(C)** Summary of optically-evoked GABA response properties in WT and Q175 SPNs at 2 (WT *n* = 21, 10 mice; Q175 *n* = 22, 10 mice), 8 (WT *n* = 20, 15 mice; Q175 *n* = 16, 11 mice) and 12 months (WT *n* = 16, eight mice; Q175 *n* = 23, 13 mice). Significant differences were determined using two-way ANOVAs and appropriate *post hoc* analyses, where *p < 0.05 and **p < 0.01.

In order to determine the relative contribution of PV- and SOM-expressing interneurons to IPSCs, we recorded SPNs from WT and Q175 mice that expressed ChR2-eYFP in the two interneuron populations (Figures 2A1,A2, 3A1,A2). We examined three groups of mice: 2 months (range 2–3 months), 8 months (range 8–9 months), and 12 months (range 12–15 months; numbers of mice and cells are in figure legends). At 2 months, heterozygous Q175 mice are not behaviorally symptomatic, while at 8 and 12 months, these mice show noticeable weight loss and deficits on motor-related tasks (Heikkinen et al., 2012; Menalled et al., 2012; Smith et al., 2014). It is also at these later ages when increases in inhibitory synaptic events have been observed in striatal SPNs (Indersmitten et al., 2015). A summary of SPN membrane properties at the three different ages is shown in Table 1. We observed a significant increase in input resistance at 8 and 12 months. This increase was also shown previously in Q175 SPNs (Indersmitten et al., 2015). Optical activation of PV interneurons in the presence of glutamate receptor antagonists (10 μM NBQX and 50 μM APV) induced IPSCs in 75%–80% of recorded cells (Figure 2B). Lack of responses in a small percentage of SPNs could be due to the inability of ChR2 to induce action potentials in some PV-expressing interneurons (Szydlowski et al., 2013). However, we observed that non-responding SPNs were generally located in regions of slices where the number of opsin-expressing PV cells was either very low or nonexistent. This observation is in line with recent studies demonstrating that FSIs target exclusively striatal SPNs within close proximity (Straub et al., 2016). Therefore, we selected recordings only from SPNs that were within ~150 μm from PV-expressing interneurons. IPSCs were similar in amplitude and area between genotypes at all ages (Figures 2B,C). There was a trend for faster IPSC decay times in Q175 SPNs at 8 months compared to WTs while at 12 months, response decay times were significantly faster (*F*_(1,112)_ = 7.17, *p* = 0.009 for genotype; Holm-Sidak *post hoc* analyses: *p* = 0.006 for Q175 vs. WT at 12 months). Interestingly, *post hoc* analyses indicated the peak amplitudes of evoked responses in 12 months WT SPNs were significantly smaller than the amplitude of responses in 2-month WTs (*p* = 0.02), suggestive of reduced connectivity between PV-expressing interneurons and SPNs with increased age (Figure 2C).

**Table 1:** Cell membrane properties of spiny projection neurons (SPNs) in wildtype (WT), Q175 and R6/2 mice.

	Capacitance (pF)	Input resistance (MΩ)	Time constant (ms)
2 months			
WT (*n* = 47)	147.6 ± 6.3	49.8 ± 2.9	2.1 ± 0.08
Q175 (*n* = 49)	143.6 ± 5.1	55.0 ± 3.0	2.1 ± 0.08
8 months			
WT (*n* = 38)	132.6 ± 4.4	64.4 ± 3.6	2.2 ± 0.08
Q175 (*n* = 33)	125.1 ± 5.7	84.9 ± 6.7**	2.1 ± 0.08
12 months			
WT (*n* = 40)	97.4 ± 6.1	78.1 ± 6.1	1.8 ± 0.10
Q175 (*n* = 47)	88.7 ± 3.8	107.5 ± 6.8**	1.8 ± 0.08
60 days			
WT (*n* = 38)	118.8 ± 6.7	66.0 ± 8.3	1.8 ± 0.1
R6/2 (*n* = 53)	80.0 ± 1.9****	161.9 ± 9.1****	1.5 ± 0.05**

Group means ± SEMs are reported. Comparisons for each membrane property were made between WT and Q175 or R6/2 cells at each age group. Significant differences were determined using Student’s t-tests where **p < 0.01 and ****p < 0.0001.

**Figure 3:**
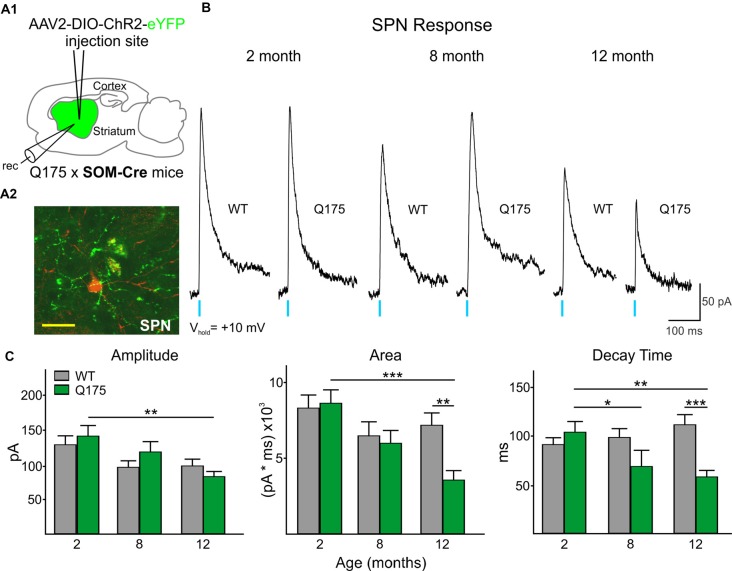
Optical activation of SOM-expressing interneurons in Q175 and WT mice at three ages.** (A1)** Schematic of AAV injection site and recording area in Q175xSOM-Cre mice. **(A2)** Confocal z-stack image of a biocytin-filled SPN (red) that was recorded from a 12 month-old Q175xSom-Cre mouse. SOM-expressing interneuron processes expressing ChR2-eYFP are seen surrounding the recorded cell. Scale bar = 20 μm. **(B)** Sample traces of evoked GABAergic responses in WT and Q175 SPNs following optical activation of SOM-expressing interneurons. **(C)** Summary of optically-evoked γ-aminobutyric acid (GABA) response properties in WT and Q175 SPNs at 2 (WT *n* = 26, 11 mice; Q175 *n* = 27, 10 mice), 8 (WT *n* = 18, seven mice; Q175, *n* = 17, nine mice) and 12 months (WT *n* = 30, 13 mice; Q175, *n* = 34, nine mice). Significant differences were determined using Two-way ANOVAs and appropriate *post hoc* analyses, where *p < 0.05, **p < 0.01 and ***p < 0.001.

All SPNs responded to optical stimulation of SOM-expressing interneurons. However, the amplitude and area of responses were significantly smaller than those evoked by activation of PV-expressing interneurons (compare Figures 3B, 2B; Amplitude: 520.6 ± 29.4 pA for all PV-expressing-induced IPSCs, *n* = 118 vs. 116.0 ± 5.4 pA for all SOM-expressing-induced IPSCs, *n* = 142; *t*_(258)_ = 14.78, *p* < 0.001. Area: 32877.6 ± 2244.1 pA × ms for all PV-expressing-induced IPSCs vs. 6816.5 ± 367.1 pA × ms for SOM-expressing-induced IPSCs; *t*_(258)_ = 12.50, *p* < 0.001). This interneuron subtype-dependent difference in evoked response size was similar to that previously observed in symptomatic R6/2 mice (Cepeda et al., 2013), a rapidly progressing transgenic mouse model that better replicates the juvenile form of HD in humans (Mangiarini et al., 1996; Lee et al., 2013). Also similar to observations in R6/2 SPNs, we found no genotype-specific differences in SOM-mediated response amplitudes in Q175 and WT SPNs at any age examined (Figures 3B,C). However, the response area was reduced in Q175 mice compared to WTs at 12 months (*F*_(2,136)_ = 3.33, *p* = 0.039 for genotype × age; Holm-Sidak *post hoc* analyses: *p* = 0.002 for Q175 vs. WT at 12 months), due to a significantly faster decay time of the evoked response (*F*_(2,136)_ = 7.45, *p* < 0.001 for genotype × age; Holm-Sidak *post hoc* analyses: *p* < 0.001 for Q175 vs. WT at 12 month). Additionally, there was a trend for faster decay times in Q175 SPNs at 8 months (*p* = 0.05). Faster decay times in responses evoked by activation of SOM-expressing interneurons is similar to the progressive changes in decay time seen in responses evoked by activation of PV-expressing interneurons in SPNs from symptomatic animals (Figure 2C and Supplementary Table S1).

### Effects of SOM-Expressing Interneuron Silencing on SPN Spontaneous IPSCs

**Figure 4:**
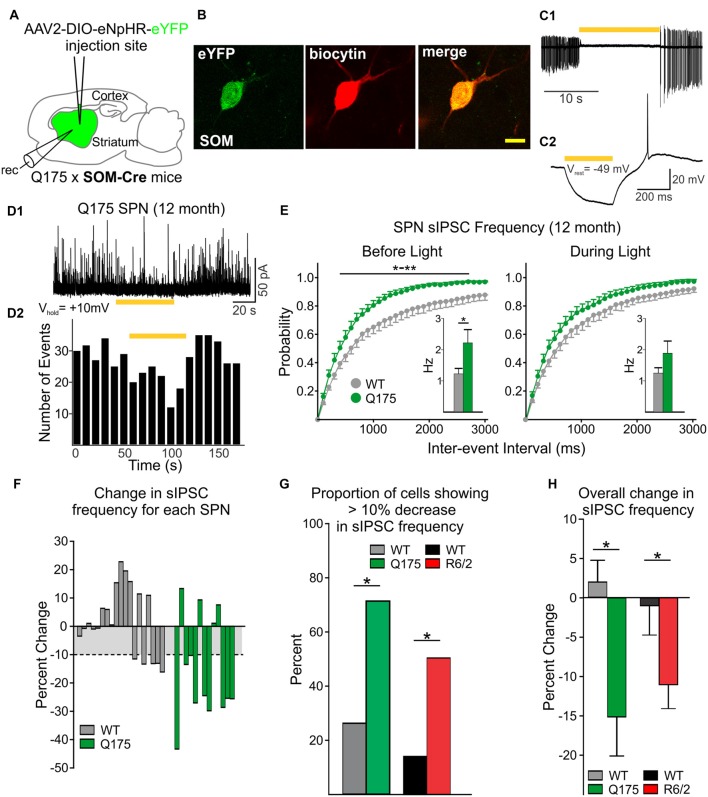
Reduced inhibitory input to SPNs is induced by silencing SOM-expressing interneurons.** (A)** Schematic of AAV injection and recording sites used for silencing experiments. **(B)** Confocal image of a biocytin-filled SOM-expressing interneuron (red) that also expresses eYFP. The cell was recorded from a 12 month old Q175xSOM-Cre mouse. Scale bar = 10 μm. **(C1)** Recording from a Q175 SOM-expressing interneuron in cell-attached mode. Most SOM interneurons are constitutively active at rest with firing frequencies of 1–10 Hz. During yellow light activation of enhanced *Natronomonas pharaonis* halorhodopsin (eNpHR; bar, 585 nm, 1 mW), the cell was silenced. **(C2)** Yellow light illumination (bar, 0.08 mW) hyperpolarizes SOM-expressing interneurons. **(D1)** Sample trace of spontaneous IPSCs (sIPSCs) recorded in an SPN from a 12 month-old Q175 mouse. Activation of eNpHR in SOM-expressing interneurons (bar, 1 mW), decreased sIPSC frequency in a subset of SPNs. **(D2)** Frequency-time histogram of recorded events in the SPN shown in D1 illustrates the decrease in sIPSC frequency during yellow light illumination. **(E)** Cumulative inter-event interval distribution plots of SPN sIPSCs before (left) and during (right) yellow light-induced suppression of SOM-expressing interneurons. Insets show the average sIPSC frequencies for each genotype. **(F)** Plots showing the percent change in sIPSC frequencies for each recorded SPN during yellow light. For WT cells that displayed a reduction in sIPSC frequency during yellow light, the mean percent change was ~10% (gray area).** (G)** Summary of the percent of SPNs that responded with a decrease (>10%) in sIPSC frequency. Proportionately, more SPNs from symptomatic Q175 (12 months) and R6/2 (60 days) mice showed a decrease in frequency compared to WT SPNs (Fisher exact test: *p* = 0.029 and *p* = 0.024, for Q175 and R6/2 vs. WT, respectively). **(H)** The average percent change (decrease) in sIPSC frequencies when SOM interneurons were silenced was greater in Q175 and R6/2 SPNs compared to WT SPNs. Significant differences were determined using Student’s t-test or two-way ANOVAs followed by appropriate *post hoc* analyses, where *p < 0.05 and **p < 0.01.

The frequency of spontaneous GABA synaptic currents in SPNs gradually increases with progression of the phenotype in several genetic mouse models of HD (Cepeda et al., 2004; Dvorzhak et al., 2013; Indersmitten et al., 2015). As LTS interneurons are one of the few classes of spontaneously active GABAergic interneurons in striatum and the increase in sIPSCs is action potential-dependent (Cepeda et al., 2004; Dvorzhak et al., 2013; Indersmitten et al., 2015), we reasoned that one possible source of increased spontaneous GABA synaptic activity in SPNs in the mouse models could be increased firing of LTS interneurons (Cepeda et al., 2013). Utilizing optogenetics, we selectively silenced this class of interneuron by expressing (eNpHR) in 12-month WT and Q175 SOM-Cre mice (six mice for each genotype; Figures 4A,B). Exposure to yellow light (1 mW) completely abolished spontaneous firing of SOM interneurons and produced a hyperpolarization of the cell membrane (Figures 4C1,C2). Notably, in a subset of SPNs, continuous yellow light (1 mW, 60 s) also significantly reduced the frequency of sIPSCs (Figures 4D1,D2), such that sIPSC frequencies from Q175 SPNs that were significantly increased compared to WTs (2.21 ± 0.4 Hz for Q175, *n* = 13 vs. 1.22 ± 0.2 Hz for WT, *n* = 19; *t*_(30)_ = 2.44, *p* = 0.021) were no longer significantly different and became similar to WTs (1.88 ± 0.4 Hz for Q175 vs. 1.24 ± 0.2 Hz for WT; *t*_(30)_ = 1.67, *p* = 0.105; Figure 4E, inset). Cumulative inter-event interval distribution plots also show sIPSC frequencies in SPNs during yellow light-induced silencing of SOM-expressing interneurons became more similar to that of WTs (Before Light: *F*_(40,1200)_ = 2.38, *p* < 0.001 for genotype × interval, Holm-Sidak *post hoc* analyses: *p* = 0.004–0.044 for intervals 400–2,700 ms. During Light: *F*_(40,1200)_ = 0.786, *p* = 0.828 for genotype × interval). The percent change of sIPSC frequencies during yellow light was greater in Q175 SPNs than in WTs (Figure 4F). We determined that the proportion of Q175 SPNs that responded to optical silencing of SOM-expressing interneurons with a reduction in sIPSC frequency greater than 10% was higher than the percentage observed in WTs (69% or 9/13 for Q175 SPNs vs. 26% or 5/19 for WTs; Fisher exact test: *p* = 0.029; Figure 4G), and for these SPNs, the average percent decrease was larger in Q175s (25.4 ± 3.2% decrease for Q175s vs. 13.4 ± 0.8% decrease for WTs; *t*_(12)_ = 2.73, *p* = 0.018). Thus, the overall change in sIPSC frequency during inhibition of SOM-expressing interneurons for all recorded SPNs was greater in Q175s than in WTs (−15.14 ± 5.0% in Q175 SPNs vs. 1.94 ± 2.7%; *t*_(30)_ = 3.24, *p* = 0.003; Figure 4H). We also used the same approach to determine if SOM-expressing interneurons contribute towards increased inhibition in the R6/2 mouse model of HD (seven and nine mice for WT and R6/2 mice, respectively). Here, we observed similar results to those of Q175 SPNs. Silencing SOM-expressing interneurons resulted in a greater proportion of R6/2 SPNs displaying a reduction (>10%) in sIPSC frequency (49% or 16/33 for R6/2 SPNs vs. 17% or 4/23 for WTs, Fisher exact test: *p* = 0.024; Figure 4G). Additionally, the overall change in average sIPSC frequency was greater in R6/2 SPNs than in WTs during yellow light (−11.0 ± 2.8% change for R6/2 s vs. −0.9 ± 3.9% change for WTs; *t*_(54)_ = 2.11, *p* = 0.04; Figure 4H). These data provide evidence that SOM-expressing interneurons are one of the main sources of increased GABAergic activity in SPNs of HD mouse models.

### Increased Frequency of sIPSCs Onto SPNs Is Not a Result of Altered GABA_B_ Signaling

In order to gain further insight into the mechanism underlying the increased inhibitory input onto SPNs, we investigated whether there were genotype-specific alterations in receptors that are predominantly located at presynaptic sites and that have been shown to modulate neurotransmitter release. First, we examined the effects of activating GABA_B_ receptor signaling. Activation of GABA_B_ receptors on presynaptic terminals was previously shown to decrease neurotransmitter release, particularly at glutamatergic corticostriatal terminals (Calabresi et al., 1991; Nisenbaum et al., 1993; Kupferschmidt and Lovinger, 2015). Furthermore, GABA release from nearby SPNs and neurogliaform-NPY interneurons can activate presynaptic GABA_B_ receptors on excitatory terminals (Logie et al., 2013). Thus, we examined whether activation of GABA_B_ receptors on PV- and SOM-expressing interneurons affects neurotransmitter release and whether such presynaptic GABA_B_ receptor signaling is altered in Q175 mice.

**Figure 5:**
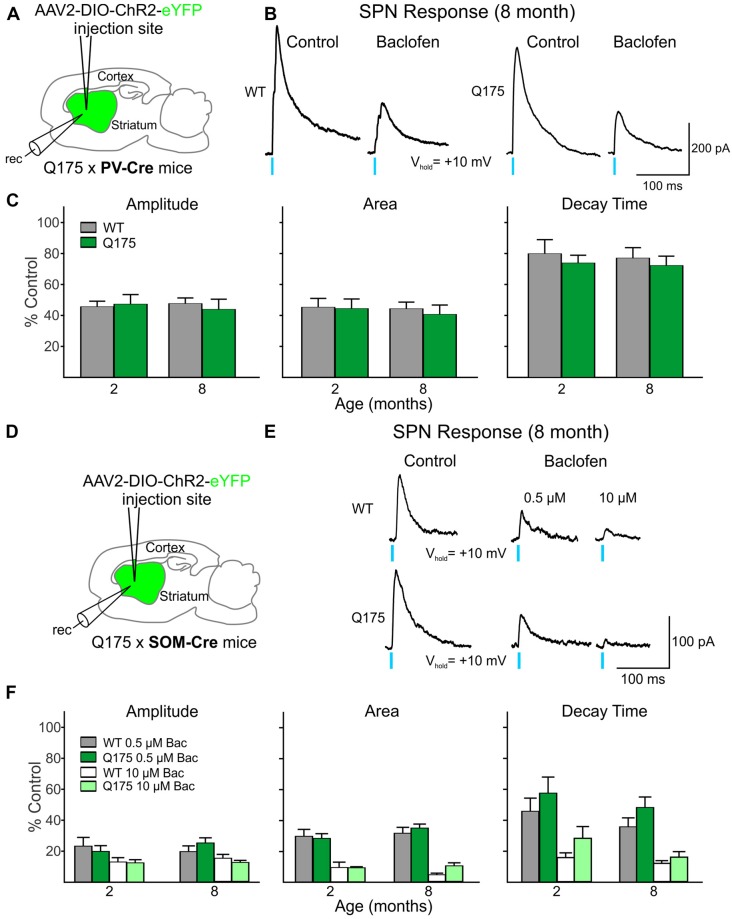
Activation of GABA_B_ receptors yielded no significant genotype-dependent alterations in PV- or SOM-expressing interneuron-induced IPSCs in Q175 SPNs.** (A)** Schematic of AAV injection/recording site and mouse line used for the data shown in **(B,C)**. **(B)** Sample traces of optically-evoked, GABA responses mediated by activating PV-expressing interneurons in WT and Q175 SPNs before (Control) and after 20 min incubation with Baclofen (Bac, 10 μM). **(C)** Summary of the effects of GABA_B_ receptor activation in WT, presymptomatic Q175 (2 months; WT *n* = 7 and Q175 *n* = 12; four and five mice, respectively) and symptomatic Q175 (8 months; WT *n* = 7 and Q175 *n* = 8; six and five mice, respectively) SPNs. Average response properties (amplitude, area and decay time) as a percentage of the Control response. **(D)** Schematic of AAV injection/recording site and mouse line used for the data shown in **(E,F)**. **(E)** Sample traces of optically-evoked, GABA responses mediated by activating SOM-expressing interneurons in WT and Q175 SPNs before (Control) and after 20 min incubation with Bac (0.5 μM followed by subsequent incubation with 10 μM). **(F)** Summary of the effects of GABA_B_ receptor activation by Bac in 2- and 8-month WT and Q175 SPNs (2 months: WT *n* = 11 and Q175 *n* = 9; eight and six mice, respectively; eight month: WT *n* = 11 and Q175 *n* = 12; six mice for each genotype.

We observed a reduction in the amplitude, area and decay time of IPSC responses induced by stimulation of PV-expressing interneurons in both Q175 and WT SPNs following incubation with the GABA_B_ receptor agonist, baclofen (Figures 5A–C). The extent of this reduction was similar in both genotypes at 2 and 8 months (numbers of mice and cells are in the figure legend). We also observed a similar pattern to activation of SOM-expressing interneuron-induced IPSCs in SPNs. Baclofen reduced similarly the amplitude, area and decay time of these responses regardless of the genotype and age (Figures 5D–F). Interestingly, the sensitivity of this baclofen-mediated reduction differed between the two interneuron types. The effective concentration of baclofen required to produce significant reductions in PV-expressing interneuron-induced responses was 10 μM. At this concentration, the amplitude decreased 56.1 ± 6.5% and 52.3 ± 3.6% for 8-month-old Q175 and WT SPNs, respectively. At 10 μM the percent reduction of SPN response amplitudes after activating SOM-expressing interneurons was 87.7 ± 1.3% in 8-month-old Q175s and 85.0 ± 2.5% in WTs at the same age. Baclofen at a concentration of 0.5 μM had a negligible effect on PV-expressing interneuron-induced responses in SPNs (data not shown). These data suggest that while the sensitivity of GABA_B_ receptor-activation differs between responses evoked by activation of the two interneuron types, GABA_B_ receptor signaling between the genotypes remains unaltered for responses in SPNs evoked by either activation of PV- or SOM-expressing interneurons.

### Reduced CB1 Receptor Modulation of GABA Responses Evoked by SOM- but Not by PV-Expressing Interneurons

**Figure 6:**
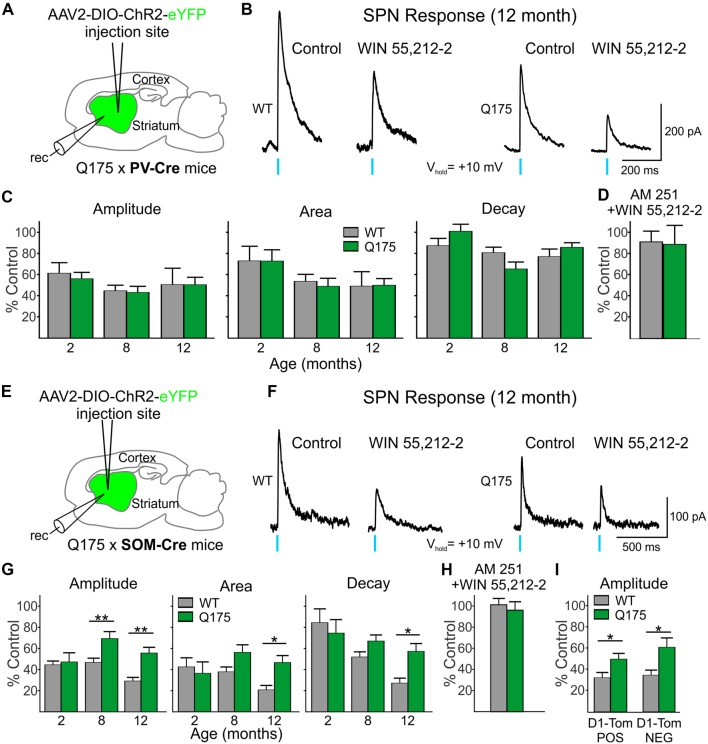
Reduced effect on GABA responses in Q175 SPNs by the cannabinoid type 1 (CB1) receptor agonist WIN 55,212-2 was observed when activating SOM- but not PV-expressing interneurons.** (A)** Schematic of AAV injection/recording site and mouse line used for the data shown in **(B–D)**. **(B)** Sample traces of optically-evoked, PV-expressing interneuron-induced GABA responses in WT and Q175 SPNs before (Control) and at the end of 20 min incubation with WIN 55,212-2 (3 μM). **(C)** Summary of the effects of CB1 activation in WT, presymptomatic Q175 (2 months) and symptomatic Q175 (8 and 12 months) SPNs. Average response properties (amplitude, area and decay time) are reported as a percentage of the Control response. **(D)** The effects of co-application of WIN-55,212-2 and the CB1 antagonist AM 251 (3 μM, 20 min) on GABA responses in WT and Q175 SPNs (2 months) following pre-incubation with AM 251. **(E)** Schematic of AAV injection/recording site and mouse line used for the data shown in **(F–I)**. **(F)** Sample traces of activation of SOM-expressing interneuron-induced GABA responses in WT and Q175 SPNs before (Control) and at the end of a 20 min incubation with WIN 55,212-2 (3 μM). **(G)** Summary of the effects of CB1 receptor activation on WT and Q175 SPNs from 2-, 8- and 12-month-old mice. **(H)** The effects of co-application of WIN-55,212-2 and AM 251 (3 μM) on GABA responses in WT and Q175 SPNs (8 months) following pre-incubation with AM 251. **(I)** The effects of presynaptic CB1 receptor activation by WIN 55,212-2 on GABA response amplitudes in SPNs from 12-month-old WT and symptomatic Q175 D1-Tom POS and D1-Tom NEG SPNs. Significant differences were determined using two-way ANOVAs and appropriate *post hoc* analyses, where *p < 0.05 and **p < 0.01.

We next examined the CB1 receptor-mediated modulation of IPSCs evoked in SPNs by optical activation of PV-expressing and SOM-expressing GABAergic interneurons (Figure 6). Control responses to activation of PV-expressing interneurons were first recorded in SPNs in the presence of glutamate receptor antagonists (10 μM NBQX and 50 μM APV) alone (Figure 6B). After a 20 min exposure to the CB1 receptor agonist, WIN 55,512-2 (3 μM), the peak amplitudes of evoked IPSCs decreased similarly in Q175 and WT SPNs [55.7% of control in SPNs from Q175 mice (*n* = 12, six mice) and to 60.9% in WT mice (*n* = 11, five mice)] at 2 months (Figure 6C). We also observed no genotype-specific differences in response areas or decay times. WIN 55,512-2-mediated decreases in evoked IPSC response amplitude and area were greater at 8 and 12 months (8 months: *n* = 11, nine mice for Q175s and *n* = 16, seven mice for WTs; 12 months: *n* = 10, eight mice for Q175 and *n* = 11, three mice for WT) than at 2 months but again, no significant differences between genotypes were observed. Additionally, no differences were observed in the response decay times at 8 and 12 months.

Activation of CB1 receptors with WIN 55,212-2 also reduced current responses in SPNs evoked by activation of SOM-expressing interneurons in both Q175 and WT mice (Figures 6E–I). At 2 months, there were no genotypic differences in optically-evoked IPSC responses following a 20 min bath application of WIN 55,212-2 (*n* = 12 for Q175s and *n* = 11 for WTs; five mice for each genotype; Figure 6G). However, at 8 months, the effect of WIN 55,212-2 on response amplitude was significantly smaller in SPNs from Q175 mice than in those from WTs (*F*_(1,67)_ = 17.18, *p* < 0.001 for genotype; Holm-Sidak *post hoc* analyses: *p* = 0.002; *n* = 12 and seven mice for Q175s and *n* = 15 and eight mice for WTs). This reduced effect of WIN 55,212-2 on response amplitude was maintained in Q175 mice at 12 months (*p* = 0.003; *n* = 10 and four mice for Q175s and *n* = 11 and six mice for WTs). Similarly, average IPSC response areas were reduced but to a lesser extent in 8-month (*p* = 0.057) and 12-month Q175 SPNs (*F*_(1,67)_ = 9.15, *p* < 0.004 for genotype; Holm-Sidak *post hoc* analyses: *p* = 0.002). The difference between mean decay times in 12 month-old SPNs following WIN 55,212-2 treatment also was statistically significant (*F*_(1,67)_ = 6.30, *p* = 0.015 for genotype; Holm-Sidak *post hoc* analyses: *p* = 0.003). If the modulation of evoked IPSCs through activation of CB1 receptors were postsynaptic, one might expect effects to be similar regardless of the type of interneuron releasing GABA. However, WIN 55,212-2 differentially affected SPN responses to activation of PV- and SOM-expressing interneurons, thus indicating that the site of action of WIN 55,212-2 primarily resides at CB1 receptors on presynaptic terminals.

Since expression of CB1 receptors on the terminals of SOM-expressing interneurons in the striatum has not previously been reported in an HD mouse model using electrophysiological techniques, we also investigated the effects of CB1 receptor activation in the R6/2 mouse model. Because Horne et al. (2013) reported reduced CB1 receptors in NPY/nNOS-positive cells in R6/2 mice previously, we chose this model to compare with findings in Q175 mice. In the striatum, there are two types of NPY cells—either those that co-express nNOS or not (Ibáñez-Sandoval et al., 2011). The NPY/nNOS-positive cells are the majority of the two and correspond to the LTS interneurons. We expressed ChR2 in WT and symptomatic (60 days) R6/2 mice and determined the effects of WIN 55,212-2 on responses evoked by activation of SOM-expressing interneurons in SPNs. Similar to the observation in 12-month Q175 SPNs, the effect of CB1 receptor activation was reduced on optically-evoked GABA responses in SPNs from R6/2 mice. There was a genotype-dependent difference with WIN 55,212-2 on the amplitude of responses (50.1 ± 6.3% of control for R6/2s, *n* = 14 and eight mice vs. 32.7 ± 3.7% of control for WTs, *n* = 11 and seven mice; *t*_(23)_ = 2.21, *p* = 0.037), as well as on response areas (46.7 ± 5.1% of control for R6/2s vs. 25.7 ± 3.6% for control in WTs; *t*_(23)_ = 3.14, *p* = 0.005) and decay times (67.5 ± 4.8% of control for R6/2 s vs. 44.8 ± 4.7% of control for WTs; *t*_(23)_ = 3.34, *p* = 0.003; data not illustrated). Thus, CB1 receptor activation on the presynaptic terminals of SOM interneurons modulates GABA responses in SPNs to a lesser degree in both symptomatic Q175 and R6/2 mice compared to WTs.

To test the specificity of the effects of WIN 55,212-2, we pre-incubated slices from WT and symptomatic (8 month) Q175 mice with the CB1 receptor antagonist AM 251 (3 μM). Peak amplitudes of IPSCs evoked in SPNs by optical stimulation of SOM-expressing interneurons were unaltered by treatment with AM 251 (94.4 ± 11.6% of control for Q175 SPNs, *n* = 4 and 99.5 ± 2.2% of control for WT SPNs, *n* = 4). Following incubation with AM 251, a combination of WIN 55,212-2 and AM 251 was applied for 20 min. AM 251 effectively blocked the previously observed effects of WIN 55,212-2 on the response amplitudes in cells of both genotypes (96.1 ± 7.9% of control for Q175 SPNs and 101.2 ± 5.9% of control for WT SPNs; Figure 6H). The same selectivity experiment was performed on 2 month-old Q175 and WT PV-Cre mice. No significant changes in the response amplitudes were observed in Q175 and WT SPNs when WIN-55,212-2 was co-applied compared to the amplitudes of control responses in AM 251 alone (96.6 ± 19.5% of control for Q175 SPNs, *n* = 3 and 99.2 ± 11.0% of control for WT SPNs, *n* = 4; Figure 6D). These results provide evidence that the reductions seen in SOM- and PV-induced IPSCs after incubation with WIN-55,212-2 were due to selective activation of CB1 receptors.

Previous work in other HD mouse models has shown inhibitory input onto D2 receptor-expressing (D2), indirect pathway SPNs is increased compared to inhibitory input onto D1 receptor-expressing (D1), direct pathway SPNs (Andre et al., 2011; Cepeda et al., 2013). Since evoked responses in SPNs from activation of SOM-expressing interneurons are less affected by WIN 55,212-2 in symptomatic Q175 SPNs compared to WTs, we tested whether or not this effect is differential for the two types of SPNs. We crossed SOM-Cre mice with a Q175 mouse line that expresses tdTomato in D1 receptor-containing SPNs. We expressed ChR2 in SOM-expressing interneurons and recorded evoked IPSCs in D1-tdTomato positive (D1-Tom POS) and negative (D1-Tom NEG) SPNs from both Q175 and WT mice (seven and six mice, respectively) at 8 months of age. Peak amplitudes of optically-evoked responses were statistically similar in D1-Tom POS and D1-Tom NEG cells in both genotypes (Q175: 83.1 ± 12.1 pA in D1-Tom POS and 86.8 ± 11.6 pA in D1-TOM NEG cells, where *n* = 10 and 10 cells, respectively; WT: 111.2 ± 21.6 pA in D1-Tom POS and 100.8 ± 14.7 pA in D1-TOM NEG cells, where *n* = 11 and 10 cells, respectively). After treatment with WIN 55,212-2 for 20 min, both D1-Tom POS and D1-TOM NEG responses were reduced similarly, although the amplitude of evoked IPSCs in Q175 SPNs of both types was less affected than SPNs in WT mice (*F*_(1,37)_ = 13.1, *p* < 0.001 for genotype; Holm-Sidak *post hoc* analyses for genotype effects: *p* = 0.045 and *p* = 0.004 for D1-tom POS and NEG, respectively; Figure 6I). Response areas also were less affected in Q175s for both types of SPNs compared to WTs (data not illustrated), although the difference was only statistically significant for D1-Tom POS cells (D1-Tom POS: 46.8 ± 7.3% of control for Q175s vs. 25.2 ± 6.7% of control for WTs; D1-Tom NEG: 47.7 ± 7.0% of control for Q175s vs. 29.9 ± 6.8% of control for WTs; *F*_(1,37)_ = 8.30, *p* = 0.007 for genotype; Holm-Sidak *post hoc* analyses for genotype effects: *p* = 0.030 and *p* = 0.076 for D1-tom POS and NEG, respectively). No significant differences were observed for response decay times for either cell type (D1-Tom POS: 55.7 ± 6.1% of control for Q175s vs. 44.0 ± 5.4% of control for WTs; D1-Tom NEG: 61.0 ± 7.7% of control for Q175s vs. 52.8 ± 7.2% of control for WTs; *F*_(1,37)_ = 2.306, *p* = 0.137; data not illustrated). These data further support our conclusion that CB1 receptors, specifically on the presynaptic terminals of SOM-expressing interneurons, are less effective at reducing the inhibition to SPNs in mouse models of HD.

To investigate whether or not altered CB1 receptors on D1 receptor-expressing SPN terminals contribute to increased inhibitory events in symptomatic Q175 mice, we examined the effects of WIN 55,212-2 on evoked IPSCs in SPNs following activation of D1 receptor-expressing SPN terminals. Here, we selectively expressed ChR2 in 13–15 month-old Q175 and WT mice that were crossed with D1-Cre mice (six and three mice, respectively). Recordings were performed only in SPNs within close proximity to the striatal injection site and that did not express ChR2. We considered these SPNs as D2 receptor-containing since they lacked opsin expression. We found no significant genotype-dependent alterations in optically-evoked IPSC response amplitudes, areas or decay times (Amplitude: 54.2 ± 7.5% of control for Q175s vs. 47.9 ± 3.7% of control for WTs, *p* = 0.457; Area: 42.7 ± 6.8% of control for Q175s vs. 39.1 ± 4.8% of control for WTs, *p* = 0.677; Decay time: 73.4 ± 6.5% of control for Q175s vs. 79.6 ± 8.7% of control for WTs, *p* = 0.575. *n* = 9 cells each). These results suggest CB1 receptor signaling is not differentially affected at Q175 D1 receptor-expressing SPN terminals in WT and Q175 mice and thereby does not contribute to the increase in inhibitory synaptic events observed in D2 receptor-expressing SPNs in HD mouse models.

### CB1 Receptor Signaling Is Affected at Corticostriatal Synapses in Symptomatic Q175 Mice

**Figure 7:**
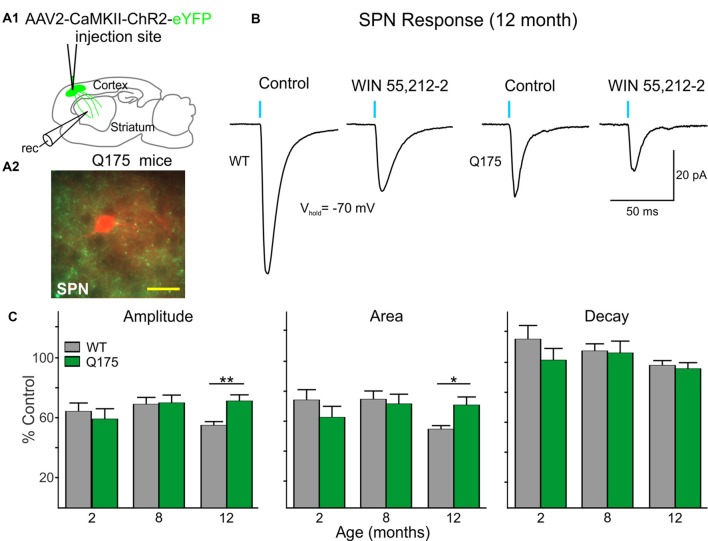
Excitatory postsynaptic currents (EPSCs) from the cortex are reduced in Q175 SPNs recorded in fully symptomatic mice and are less sensitive to modulation by WIN 55,212-2 compared to WT SPNs.** (A1)** Schematic of AAV injection site and recording area in Q175 mice for experiments examining CB1 receptor-mediated modulation of excitatory cortical inputs onto SPNs. **(A2)** Confocal z-stack image of a biocytin-filled SPN (red) that was recorded from an 8 months-old Q175 mouse. Corticostriatal processes expressing ChR2-eYFP are seen surrounding the recorded cell. Scale bar = 20 μm. **(B)** Sample traces of optically-evoked, cortical-induced glutamatergic responses in WT and Q175 SPNs before (Control) and at the end of 20 min incubation with WIN 55,212-2 (3 μM). **(C)** Summary of the effects of WIN 55,212-2 (3 μM) on optically-evoked EPSCs from WT and Q175 SPNs from 2-, 8- and 12-month-old mice. Average response properties (amplitude, area and decay time) are reported as a percentage of the Control response. Significant differences were determined using two-way ANOVAs and appropriate *post hoc* analyses, where *p < 0.05 and **p < 0.01.

Since it has been shown that activation of CB1 receptors on excitatory cortical afferents can modulate glutamate release in the striatum (Gerdeman and Lovinger, 2001) we also investigated whether CB1 receptor-mediated signaling on corticostriatal terminals is progressively affected in Q175 mice. ChR2 driven by the CaMKII promoter was injected in the right M1 cortical region of 2, 8 and 12 month-old Q175 and WT mice (Figure 7A1). Slices were decorticated and optically-evoked EPSCs were recorded in SPNs in the presence of the GABA_A_ receptor antagonist, bicuculline (BIC, 10 μM; Figure 7B). No genotype-specific differences in the amplitude or area of evoked EPSCs were observed until 12 months. At this age, the evoked EPSCs in Q175 SPNs (*n* = 22) were smaller in amplitude (283.8 ± 50.6 pA for Q175 SPNs vs. 533.4 ± 52.4 pA for WTs; *F*_(1,107)_ = 4.19, *p* = 0.043 for genotype; Holm-Sidak *post hoc* analyses: *p* = 0.004) and there was a trend for smaller area (5539.5 ± 1290.6 pA × ms for Q175 SPNs vs. 10388.2 ± 1262.3 pA × ms for WTs, *p* = 0.096 for genotype), but decay times were similar to WTs (*n* = 24, 23.3 ± 2.4 ms for Q175 SPNs and 24.4 ± 1.7 ms for WTs). Similarly, genotype-specific alterations in CB1-mediated modulation of corticostriatal inputs were not apparent until 12 months (Figure 7C). Evoked corticostriatal EPSCs in Q175 SPNs (*n* = 21) were less sensitive to CB1 modulation compared to EPSCs in WTs (*n* = 22; amplitude: *F*_(2,95)_ = 3.13, *p* = 0.043 for genotype × age; Holm-Sidak *post hoc* analyses: *p* = 0.005; area: *F*_(2,94)_ = 3.37, *p* = 0.039; Holm-Sidak *post hoc* analyses: *p* = 0.025). The reduced CB1 effect on Q175 SPN IPSCs from SOM-expressing interneurons appears to precede the CB1 receptor-mediated alterations observed in Q175 corticostriatal projections (compare Figures 6, 7).

## Discussion

Motor impairment as a result of striatal atrophy and microcircuit dysfunction is a hallmark pathology of HD. While much attention has focused on alterations in glutamatergic signaling due to the excitotoxic nature of glutamate, changes in the corticostriatal pathway may only partially explain the progression of motor abnormalities associated with HD. Alterations in GABAergic synaptic transmission in the striatum are observed in symptomatic HD mice and are also likely to contribute to abnormal movements (Cepeda et al., 2004, 2013; Dvorzhak et al., 2013; Indersmitten et al., 2015; Garret et al., 2018; Reiner and Deng, 2018). We focused on striatal GABAergic interneurons as the main sources of increased inhibitory synaptic transmission since abnormalities in their intrinsic and synaptic properties have been observed in HD mice (Cepeda et al., 2013; Holley et al., 2019). By selectively exciting or inhibiting discrete interneuron subtypes with optogenetic techniques, we examined the synaptic contributions of PV- and SOM-expressing interneurons in the Q175 HD mouse model. Optically silencing striatal SOM-expressing interneurons, which normally fire spontaneously, reduced the activity-dependent enhancement of spontaneous GABA synaptic currents in SPNs from HD mice to levels observed in WT mice. Our results also suggest that a plausible mechanism contributing to the enhancement of GABA synaptic activity in HD mice is the downregulation of CB1 receptors located on presynaptic terminals of SOM- but not PV-expressing interneurons. In addition, we found that although the amplitude of GABA responses induced in SPNs by optical activation of PV- or SOM-expressing interneurons was not different between genotypes, the decay time was significantly faster in SPNs from HD mice. These findings offer insight into the relative impact that each of these two types of interneurons has in altering striatal output by modulating the activity of SPNs in HD.

In agreement with previous studies, we observed the average sIPSC frequency in Q175 SPNs was increased compared to WTs (Dvorzhak et al., 2013; Indersmitten et al., 2015). Since SOM-expressing but not PV-expressing interneurons are spontaneously active at rest and increased sIPSC frequency is activity-dependent, we posited that these interneurons play a pivotal role in this effect. We previously reported in Q175 mice that some LTS interneurons displayed increased action potential firing within oscillating bursts (Holley et al., 2019) thus predicting that while in this more excitable state, they may release more GABA onto SPNs. As such, we demonstrate that optical activation of halorhodopsin in SOM-expressing interneurons induced a decrease in the overall frequency of sIPSCs that was more pronounced and occurred in a greater percentage of SPNs in symptomatic Q175 and R6/2 mice than in WTs. Further, silencing SOM-expressing interneurons reduced sIPSC frequencies in Q175 SPNs to levels observed in WTs. These results support the premise that excessive activity of SOM-expressing interneurons primarily contributes to the increased GABA synaptic activity observed in SPNs in HD mouse models. Incidentally, striatal SOM levels are increased in HD patients (Aronin et al., 1983) supporting the hypothesis that increased firing of these interneurons also results in the increased release of neuroprotective factors. Although we can only surmise that the reason for the increase in SOM-expressing interneuron activity is a potential compensatory mechanism to offset the glutamatergic dysregulation observed in HD mice (Cepeda et al., 2007; Bunner and Rebec, 2016), an unintended consequence of SOM upregulation is also an increase in spontaneous, activity-dependent, GABA synaptic activity (Cepeda et al., 2004).

Since we previously observed evoked IPSCs with larger amplitudes in SPNs of R6/2 mice compared to WTs following activation of PV-expressing interneurons (Cepeda et al., 2013), we presumed that PV-expressing interneurons would also be a main contributor to increased GABA synaptic activity in the striatum of symptomatic Q175 mice (Indersmitten et al., 2015). Additionally, FSIs exhibit greater connectivity to SPNs than LTS or other neighboring SPNs (Tepper et al., 2004; Cepeda et al., 2013) and in Q175 mice the resting membrane potentials of these interneurons are more depolarized (Holley et al., 2019), thus making PV-expressing interneurons a likely candidate involved in the increased inhibition onto SPNs. However, unlike what was observed in R6/2 mice (Cepeda et al., 2013), we saw no genotype-dependent differences in evoked IPSC amplitudes after optically-stimulating either PV- or SOM-expressing interneurons in Q175 mice. This is likely due to how responsive intracellular homeostatic mechanisms within PV-expressing interneurons are in the context of a severe vs. a more slowly progressing HD environment. In a recent study from our laboratory, PV-expressing interneurons in the Q175 striatum were found to exhibit signs of degeneration as evidenced by the decreased cell membrane capacitances, smaller somatic areas and reduced neurite arborizations in these interneurons (Holley et al., 2019). Likewise, in HD patients with the adult-onset form of the disease where the progression of the disease phenotype takes decades to fully manifest, the number of PV-expressing interneurons is reduced while the number of SOM-expressing neurons remains constant (Reiner et al., 2013). Thus, PV-expressing interneurons appear to be more vulnerable over long periods of time, implying that they lack the ability to upregulate GABA synaptic activity through possible loss of synaptic connections to SPNs. It would be of interest to determine if PV-interneuron connectivity to other striatal neurons is altered in HD. Interestingly, loss of PV-expressing neurons in addition to morphological signs of degeneration have also been observed in the brains of multiple sclerosis patients and mouse models (Dutta et al., 2006; Clements et al., 2008; Falco et al., 2014).

We provide evidence that a plausible contributing mechanism underlying the overall increase in the frequency of sIPSCs induced by the hyperactivity of SOM-expressing interneurons is a dysregulation of the endocannabinoid system in these interneurons. Selectively activating SOM-expressing interneurons in the presence of WIN 55,212-2 demonstrated that CB1 receptors on the terminals of these interneurons are either reduced or less sensitive in Q175 mice, beginning at 8 months of age. The prevailing function of CB1 receptor activation is to inhibit synaptic activity and likewise, activation of CB1 receptors has been shown to suppress both excitatory and inhibitory neurotransmitter release in multiple brain regions (Szabo et al., 1998; Katona et al., 1999; Hajos et al., 2000; Gerdeman and Lovinger, 2001; Hentges et al., 2005; reviewed in Katona and Freund, 2012). In the striatum of HD patients and in mouse models, CB1 receptor mRNA transcript levels and binding of exogenous cannabimimetics is reduced (Denovan-Wright and Robertson, 2000; Glass et al., 2000; Lastres-Becker et al., 2002; McCaw et al., 2004; Menalled et al., 2012). Additionally, previous studies showed the sensitivity of striatal GABA synapses to cannabinoid receptor stimulation is severely impaired in R6/2 mice (Centonze et al., 2005). We now demonstrate that this effect is selective for SOM-, not PV-expressing interneurons in symptomatic 8-month-old mice at synapses on both direct (D1) and indirect (D2) pathway SPNs. This observation agrees with a previous study in HD showing that CB1 receptors are downregulated specifically in NPY/NOS-expressing interneurons while remaining unchanged in PV-expressing interneurons (Horne et al., 2013). We also observed decreased CB1 sensitivity to cortical inputs, but not until 12 months of age suggesting that HD-related alterations of CB1 receptors on inhibitory SOM-interneuron terminals may precede those on excitatory corticostriatal projections. However, solid evidence would require additional quantitative immunohistochemical analyses in addition to ligand binding studies.

While attempting to elucidate a mechanism behind this increased inhibitory synaptic activity in the HD striatum, we also sought to determine if interneuron-specific GABA_B_ signaling was affected in Q175 mice. Additionally, excess GABA may affect neurotransmitter release (excitatory and inhibitory) through activation of presynaptic GABA_B_ receptors. Although we observed that GABA_B_ signaling at interneuron-SPN synapses is intact in presymptomatic and symptomatic mice, we found the sensitivity of the IPSC depression differed between PV- and SOM-expressing interneuron synapses. Unlike ionotropic GABA_A_ receptors that rapidly activate upon ligand binding and can alter membrane potentials due to their permeability to Cl^−^ ions (Olsen and Sieghart, 2009), GABA_B_ receptors are coupled to intracellular G-protein signaling cascades and are able to modulate neurotransmitter release by inhibiting voltage-dependent Ca^2+^ channels (Wu and Saggau, 1995). Thus, it would seem likely that if terminals of SOM-expressing interneurons contain more GABA_B_ receptors than the terminals of PV-expressing interneurons, a GABA_B_ receptor agonist would produce greater inhibition of neurotransmitter release at these synapses. Although the density of GABA_B_ receptors on striatal interneuron terminals has not been well characterized, this does seem to be the case for SOM interneuron synapses in the rat hippocampus where immunolabeling of the GABA_B_R-1 subunit is stronger than for all other cells (Sloviter et al., 1999). Additionally, baclofen produced a greater depression of SOM-expressing interneuron-mediated IPSCs compared to those mediated by activation of PV-expressing interneurons in the dorso-medial prefrontal cortex of mice (Liu et al., 2017). Thus, we contend that the increased frequency of sIPSCs in HD mice is not dependent on dysfunctional GABA_B_ signaling at PV and SOM-expressing interneuron terminals. However, alterations in GABA_B_ receptor signaling at other interneuron or SPN synapses in HD mice cannot be excluded.

We observed that the amplitude of GABA responses evoked in SPNs by photoactivation of PV- and SOM-expressing interneurons was not affected between genotypes, but the decay time was significantly faster in cells from Q175 mice, beginning at 8 months for responses evoked by SOM-expressing interneuron activation and at 12 months for responses evoked by PV-expressing interneuron activation, although there was a trend for more rapid kinetics at 8 months as well. Non-selectively activating all GABAergic inputs with electrical stimulation also resulted in IPSC responses with similar amplitudes in Q175 and WT SPNs but displayed more rapid decay times (unpublished observation). Faster decay times of spontaneous, electrically- and pharmacologically-evoked GABA responses also were observed in slices and dissociated SPNs from R6/2 mice (Cepeda et al., 2004, 2013). Similar to observations in symptomatic Q175 mice, evoked IPSCs following activation of PV- and SOM-expressing interneurons in R6/2 mice exhibited faster decay times. It is enigmatic to observe more rapid kinetics in responses evoked by stimulating GABAergic interneurons while decay time kinetics appear similar to WTs for evoked excitatory responses in Q175 SPNs and for inhibitory responses induced by stimulation of other SPNs. Given that rapid decay times appear to be associated with mainly interneurons than other cell types in HD mice, possible explanations include alterations in mechanisms of presynaptic neurotransmitter release (Hefft and Jonas, 2005; Keros and Hablitz, 2005), differential expression of GABA transporters (Overstreet and Westbrook, 2003), modulation of postsynaptic GABA receptor subunit composition or subtypes or a combination of any of the above. Nevertheless. more rapid kinetics was shown to be associated with enhanced expression of the α1 subunit, corroborating the idea that this subunit contributes to faster IPSCs (Okada et al., 2000; Barberis et al., 2007). Incidentally, α1 subunit expression also is increased in the striatum of early and fully symptomatic HD mice (Cepeda et al., 2004; Du et al., 2017).

In the present study, we also observed a reduction in the amplitude of PV-expressing interneuron-evoked IPSCs as a function of age in WT but not in Q175 mice. This effect suggests reduced connectivity between FSIs and SPNs with age. In our previous studies looking at the effects of age on corticostriatal neurotransmission, we demonstrated an age-dependent decline of evoked excitatory responses (Cepeda et al., 1996). Here we show this effect can be generalized to PV-expressing interneuron-mediated inhibitory neurotransmission, consistent with neurochemical studies showing reduced levels of GABA transmission in old mice (see for example Duarte et al., 2014).

While the present findings point to SOM interneurons as the primary source of increased GABA synaptic activity in HD mice, other potential sources should be considered. For example, a subset of pallidal neurons project back to the striatum (Mastro et al., 2014; Abdi et al., 2015; Glajch et al., 2016). Although the main target of GPe inhibitory input is the FSIs, it is possible that a small proportion innervates MSNs (Glajch et al., 2016). In addition, long-range, PV- and SOM-mediated inhibitory cortical inputs to striatal SPNs were described recently (Rock et al., 2016; Melzer et al., 2017) and other types of striatal interneurons (e.g., TH- and calretinin-expressing interneurons) could potentially contribute to aberrant GABAergic synaptic activity in HD. The role of these inputs needs to be elucidated in future studies. In addition, although a postsynaptic mechanism is the most likely explanation for faster decay times, further experiments exploring changes in paired-pulse ratios using an opsin with a high temporal resolution, as well as changes in GABA_A_ receptor subunit composition in the striatum of Q175 mice seem warranted.

Finally, in the present study, we could not determine if differences in frequency of sIPSCs occur in SPNs belonging to the direct or indirect pathway. Our previous studies in the more severe R6/2 transgenic mouse model showed that the increase in GABA activity affects mostly MSNs of the indirect pathway (Cepeda et al., 2013). The changes in IPSC frequency observed in Q175 knock-in mice are more complex as they are age- and gene dose-dependent. In Q175^+/–^ mice the change in frequency is subtle and does not reach statistical significance, whereas, in homozygous mice (Q175^+/+^), increased frequency of sIPSCs is statistically significant (Indersmitten et al., 2015). As the present study was constrained to using heterozygous Q175 mice, this experiment could not be performed.

## Conclusions and Interpretations

The present findings demonstrate that SOM- but not PV-expressing interneurons are the main contributors to increased GABA synaptic activity observed in genetic mouse models of HD. Upregulation of SOM-expressing interneuron function is associated with loss and/or reduced sensitivity of CB1 receptors specifically on this type of interneuron. Furthermore, optogenetic silencing of SOM-expressing interneurons reestablished normal GABA synaptic activity, suggesting novel avenues to restore the excitatory/inhibitory balance lost in HD. However, if indeed SOM has neuroprotective properties, caution must be exerted not to perturb this homeostatic mechanism.

At present, it is still premature to explain why SOM-expressing interneuron activity is upregulated in HD. Although we could speculate that increased activity is a compensatory mechanism to prevent excitotoxic levels of glutamate released from cortical and thalamic terminals, previous studies have demonstrated that neurogliaform, not SOM-expressing, interneurons are able to modulate cortical inputs (Logie et al., 2013). Further, we also have to consider that in HD GABA could be excitatory, at least in the hippocampus of symptomatic mice (Dargaei et al., 2019). Interestingly, a recent study found that both GABA and glutamate can be released simultaneously onto striatal SPNs in response to photoactivation of SOM-expressing interneurons (Cattaneo et al., 2019). While the glutamatergic response is short-lived, the GABA response is persistent. In HD mouse models we demonstrated a progressive reduction in glutamatergic inputs onto SPNs (Cepeda et al., 2003). It is possible that, if indeed SOM-expressing interneuron activation is both excitatory and inhibitory, reduced excitatory input can be supplanted by intrinsic activation from SOM-expressing interneurons. Thus, at present, the most parsimonious explanation for SOM-expressing interneuron upregulation in HD is either to fulfill a neuroprotective role or to compensate for the loss of excitatory inputs.

## Ethics Statement

This study was carried out in accordance with the recommendations of the United States Public Health Service Guide for Care and Use of Laboratory Animals.

## Author Contributions

SH, LG, ML and CC designed research. SH and LG performed research. SH, LG, TK and AD analyzed data. SH, ML and CC wrote the article.

## Conflict of Interest Statement

The authors declare that the research was conducted in the absence of any commercial or financial relationships that could be construed as a potential conflict of interest.

## Abbreviations

ACSF: artificial cerebrospinal fluid
AMPA: α-amino-3-hydroxy-5-methyl-4-isoxazolepropionic acid
CAG: cytosine–adenine–guanine
CaMKII: Ca^2+^/calmodulin-dependent protein kinase II
CB1: cannabinoid receptor type 1
ChR2: channelrhodopsin-2
D1: dopamine D1 receptor type
eNpHR: enhanced *Natronomonas pharaonis* halorhodopsin
FSI: fast-spiking interneuron
GABA: γ-aminobutyric acid
HD: Huntington’s disease
LTS: low-threshold spiking (interneuron)
NMDA: N-methyl-D-aspartate
NOS: nitric oxide synthase
NPY: neuropeptide-Y
PV: parvalbumin
EPSC: excitatory postsynaptic current
IPSC: inhibitory postsynaptic current
SOM: somatostatin
SPN: spiny projection neuron
WT: wildtype.

## References

[B1] Abdi A. Mallet N. Mohamed F. Y. Sharott A. Dodson P. D. Nakamura K. C.. (2015). Prototypic and arkypallidal neurons in the dopamine-intact external globus pallidus. J. Neurosci. 35, 6667–6688. 10.1523/JNEUROSCI.4662-14.2015 25926446 PMC4412890

[B2] Albin R. L. Reiner A. Anderson K. D. Penney J. B. Young A. B. (1990). Striatal and nigral neuron subpopulations in rigid Huntington’s disease: implications for the functional anatomy of chorea and rigidity-akinesia. Ann. Neurol. 27, 357–365. 10.1002/ana.410270403 1972318

[B3] Andre V. M. Fisher Y. E. Levine M. S. (2011). Altered balance of activity in the striatal direct and indirect pathways in mouse models of Huntington’s disease. Front. Syst. Neurosci. 5:46. 10.3389/fnsys.2011.00046 21720523 PMC3118454

[B4] Aronin N. Cooper P. E. Lorenz L. J. Bird E. D. Sagar S. M. Leeman S. E.. (1983). Somatostatin is increased in the basal ganglia in Huntington disease. Ann. Neurol. 13, 519–526. 10.1002/ana.410130508 6191621

[B5] Barberis A. Mozrzymas J. W. Ortinski P. I. Vicini S. (2007). Desensitization and binding properties determine distinct α1β2γ2 and α3β2γ2 GABA_A_ receptor-channel kinetic behavior. Eur. J. Neurosci. 25, 2726–2740. 10.1111/j.1460-9568.2007.05530.x 17561840 PMC1950087

[B6] Bates G. P. Dorsey R. Gusella J. F. Hayden M. R. Kay C. Leavitt B. R.. (2015). Huntington disease. Nat. Rev. Dis. Primers 1:15005. 10.1038/nrdp.2015.5 27188817

[B7] Bolam J. P. Hanley J. J. Booth P. A. Bevan M. D. (2000). Synaptic organisation of the basal ganglia. J. Anat. 196, 527–542. 10.1046/j.1469-7580.2000.19640527.x 10923985 PMC1468095

[B8] Bunner K. D. Rebec G. V. (2016). Corticostriatal dysfunction in Huntington’s disease: the basics. Front. Hum. Neurosci. 10:317. 10.3389/fnhum.2016.00317 27445757 PMC4924423

[B9] Calabresi P. Mercuri N. B. De Murtas M. Bernardi G. (1991). Involvement of GABA systems in feedback regulation of glutamate-and GABA-mediated synaptic potentials in rat neostriatum. J. Physiol. 440, 581–599. 10.1113/jphysiol.1991.sp018726 1666654 PMC1180170

[B700] Cattaneo S. Zaghi M. Maddalena R. Bedogni F. Sessa A. Taverna S. (2019). Somatostatin-expressing interneurons co-release GABA and glutamate onto different postsynaptic targets in the striatum. bioRxiv. 10.1101/566984 Available online at: https://www.biorxiv.org/content/10.1101/566984v2. Accessed May 1, 2019.

[B10] Centonze D. Rossi S. Prosperetti C. Tscherter A. Bernardi G. Maccarrone M.. (2005). Abnormal sensitivity to cannabinoid receptor stimulation might contribute to altered γ-aminobutyric acid transmission in the striatum of R6/2 Huntington’s disease mice. Biol. Psychiatry 57, 1583–1589. 10.1016/j.biopsych.2005.03.008 15953496

[B11] Cepeda C. Cummings D. M. André V. M. Holley S. M. Levine M. S. (2010). Genetic mouse models of Huntington’s disease: focus on electrophysiological mechanisms. ASN Neuro 2:e00033. 10.1042/an20090058 20396376 PMC2850512

[B12] Cepeda C. Galvan L. Holley S. M. Rao S. P. André V. M. Botelho E. P.. (2013). Multiple sources of striatal inhibition are differentially affected in Huntington’s disease mouse models. J. Neurosci. 33, 7393–7406. 10.1523/JNEUROSCI.2137-12.2013 23616545 PMC3686572

[B13] Cepeda C. Hurst R. S. Calvert C. R. Hernández-Echeagaray E. Nguyen O. K. Jocoy E.. (2003). Transient and progressive electrophysiological alterations in the corticostriatal pathway in a mouse model of Huntington’s disease. J. Neurosci. 23, 961–969. 10.1523/JNEUROSCI.23-03-00961.2003 12574425 PMC6741903

[B14] Cepeda C. Li Z. Levine M. S. (1996). Aging reduces neostriatal responsiveness to N-methyl-D-aspartate and dopamine: an *in vitro* electrophysiological study. Neuroscience 73, 733–750. 10.1016/0306-4522(96)00056-5 8809794

[B15] Cepeda C. Starling A. J. Wu N. Nguyen O. K. Uzgil B. Soda T.. (2004). Increased GABAergic function in mouse models of Huntington’s disease: reversal by BDNF. J. Neurosci. Res. 78, 855–867. 10.1002/jnr.20344 15505789

[B16] Cepeda C. Wu N. Andre V. M. Cummings D. M. Levine M. S. (2007). The corticostriatal pathway in Huntington’s disease. Prog. Neurobiol. 81, 253–271. 10.1016/j.pneurobio.2006.11.001 17169479 PMC1913635

[B17] Chuhma N. Tanaka K. F. Hen R. Rayport S. (2011). Functional connectome of the striatal medium spiny neuron. J. Neurosci. 31, 1183–1192. 10.1523/JNEUROSCI.3833-10.2011 21273403 PMC3074638

[B18] Clements R. J. McDonough J. Freeman E. J. (2008). Distribution of parvalbumin and calretinin immunoreactive interneurons in motor cortex from multiple sclerosis post-mortem tissue. Exp. Brain Res. 187, 459–465. 10.1007/s00221-008-1317-9 18297277

[B19] Dargaei Z. Liang X. Serranilla M. Santos J. Woodin M. A. (2019). Alterations in hippocampal inhibitory synaptic transmission in the R6/2 mouse model of Huntington’s disease. Neuroscience 404, 130–140. 10.1016/j.neuroscience.2019.02.007 30797895

[B20] Denovan-Wright E. M. Robertson H. A. (2000). Cannabinoid receptor messenger RNA levels decrease in a subset of neurons of the lateral striatum, cortex and hippocampus of transgenic Huntington’s disease mice. Neuroscience 98, 705–713. 10.1016/s0306-4522(00)00157-3 10891614

[B21] Du Z. Tertrais M. Courtand G. Leste-Lasserre T. Cardoit L. Masmejean F.. (2017). Differential alteration in expression of striatal GABAAR subunits in mouse models of Huntington’s disease. Front. Mol. Neurosci. 10:198. 10.3389/fnmol.2017.00198 28676743 PMC5476702

[B22] Duarte J. M. Do K. Q. Gruetter R. (2014). Longitudinal neurochemical modifications in the aging mouse brain measured *in vivo* by 1H magnetic resonance spectroscopy. Neurobiol. Aging 35, 1660–1668. 10.1016/j.neurobiolaging.2014.01.135 24560998

[B23] Dutta R. McDonough J. Yin X. Peterson J. Chang A. Torres T.. (2006). Mitochondrial dysfunction as a cause of axonal degeneration in multiple sclerosis patients. Ann. Neurol. 59, 478–489. 10.1002/ana.20736 16392116

[B24] Dvorzhak A. Semtner M. Faber D. S. Grantyn R. (2013). Tonic mGluR5/CB1-dependent suppression of inhibition as a pathophysiological hallmark in the striatum of mice carrying a mutant form of huntingtin. J. Physiol. 591, 1145–1166. 10.1113/jphysiol.2012.241018 23230231 PMC3591720

[B25] Falco A. Pennucci R. Brambilla E. de Curtis I. (2014). Reduction in parvalbumin-positive interneurons and inhibitory input in the cortex of mice with experimental autoimmune encephalomyelitis. Exp. Brain Res. 232, 2439–2449. 10.1007/s00221-014-3944-7 24770856 PMC4055863

[B26] Garret M. Du Z. Chazalon M. Cho Y. H. Baufreton J. (2018). Alteration of GABAergic neurotransmission in Huntington’s disease. CNS Neurosci. Ther. 24, 292–300. 10.1111/cns.12826 29464851 PMC6490108

[B27] Gerdeman G. Lovinger D. M. (2001). CB1 cannabinoid receptor inhibits synaptic release of glutamate in rat dorsolateral striatum. J. Neurophysiol. 85, 468–471. 10.1152/jn.2001.85.1.468 11152748

[B28] Glajch K. E. Kelver D. A. Hegeman D. J. Cui Q. Xenias H. S. Augustine E. C.. (2016). Npas1^+^ pallidal neurons target striatal projection neurons. J. Neurosci. 36, 5472–5488. 10.1523/JNEUROSCI.1720-15.2016 27194328 PMC4871984

[B29] Glass M. Dragunow M. Faull R. L. (2000). The pattern of neurodegeneration in Huntington’s disease: a comparative study of cannabinoid, dopamine, adenosine and GABA_A_ receptor alterations in the human basal ganglia in Huntington’s disease. Neuroscience 97, 505–519. 10.1016/s0306-4522(00)00008-7 10828533

[B30] Hajos N. Katona I. Naiem S. S. MacKie K. Ledent C. Mody I.. (2000). Cannabinoids inhibit hippocampal GABAergic transmission and network oscillations. Eur. J. Neurosci. 12, 3239–3249. 10.1046/j.1460-9568.2000.00217.x 10998107

[B31] Hefft S. Jonas P. (2005). Asynchronous GABA release generates long-lasting inhibition at a hippocampal interneuron-principal neuron synapse. Nat. Neurosci. 8, 1319–1328. 10.1038/nn1542 16158066

[B32] Heikkinen T. Lehtimaki K. Vartiainen N. Puoliväali J. Hendricks S. J. Glaser J. R.. (2012). Characterization of neurophysiological and behavioral changes, MRI brain volumetry and 1H MRS in zQ175 knock-in mouse model of Huntington’s disease. PLoS One 7:e50717. 10.1371/journal.pone.0050717 23284644 PMC3527436

[B33] Hentges S. T. Low M. J. Williams J. T. (2005). Differential regulation of synaptic inputs by constitutively released endocannabinoids and exogenous cannabinoids. J. Neurosci. 25, 9746–9751. 10.1523/JNEUROSCI.2769-05.2005 16237178 PMC6725733

[B34] Holley S. M. Galvan L. Kamdjou T. Cepeda C. Levine M. S. (2019). Striatal GABAergic interneuron dysfunction in the Q175 mouse model of Huntington’s disease. Eur. J. Neurosci. 49, 79–93. 10.1111/ejn.14283 30472747 PMC8320683

[B35] Holley S. M. Joshi P. R. Parievsky A. Galvan L. Chen J. Y. Fisher Y. E.. (2015). Enhanced GABAergic inputs contribute to functional alterations of cholinergic interneurons in the R6/2 mouse model of Huntington’s disease. eNeuro 2:e0008. 10.1523/eneuro.0008-14.2015 26203463 PMC4507822

[B36] Horne E. A. Coy J. Swinney K. Fung S. Cherry A. E. Marrs W. R.. (2013). Downregulation of cannabinoid receptor 1 from neuropeptide Y interneurons in the basal ganglia of patients with Huntington’s disease and mouse models. Eur. J. Neurosci. 37, 429–440. 10.1111/ejn.12045 23167744 PMC3699342

[B37] Hsu Y. T. Chang Y. G. Chern Y. (2018). Insights into GABAAergic system alteration in Huntington’s disease. Open Biol. 8:180165. 10.1098/rsob.180165 30518638 PMC6303784

[B38] Ibáñez-Sandoval O. Tecuapetla F. Unal B. Shah F. Koós T. Tepper J. M. (2011). A novel functionally distinct subtype of striatal neuropeptide Y interneuron. J. Neurosci. 31, 16757–16769. 10.1523/JNEUROSCI.2628-11.2011 22090502 PMC3236391

[B39] Indersmitten T. Tran C. H. Cepeda C. Levine M. S. (2015). Altered excitatory and inhibitory inputs to striatal medium-sized spiny neurons and cortical pyramidal neurons in the Q175 mouse model of Huntington’s disease. J. Neurophysiol. 113, 2953–2966. 10.1152/jn.01056.2014 25673747 PMC4416625

[B40] Katona I. Freund T. F. (2012). Multiple functions of endocannabinoid signaling in the brain. Annu. Rev. Neurosci. 35, 529–558. 10.1146/annurev-neuro-062111-150420 22524785 PMC4273654

[B41] Katona I. Sperlágh B. Sík A. Käfalvi A. Vizi E. S. Mackie K.. (1999). Presynaptically located CB1 cannabinoid receptors regulate GABA release from axon terminals of specific hippocampal interneurons. J. Neurosci. 19, 4544–4558. 10.1523/JNEUROSCI.19-11-04544.1999 10341254 PMC6782612

[B42] Keros S. Hablitz J. J. (2005). Subtype-specific GABA transporter antagonists synergistically modulate phasic and tonic GABA_A_ conductances in rat neocortex. J. Neurophysiol. 94, 2073–2085. 10.1152/jn.00520.2005 15987761

[B43] Kumar U. (2008). Somatostatin in medium-sized aspiny interneurons of striatum is responsible for their preservation in quinolinic acid and N-methyl-D-asparate-induced neurotoxicity. J. Mol. Neurosci. 35, 345–354. 10.1007/s12031-008-9093-3 18483877

[B44] Kupferschmidt D. A. Lovinger D. M. (2015). Inhibition of presynaptic calcium transients in cortical inputs to the dorsolateral striatum by metabotropic GABA_B_ and mGlu2/3 receptors. J. Physiol. 593, 2295–2310. 10.1113/jp270045 25781000 PMC4457193

[B45] Lastres-Becker I. Berrendero F. Lucas J. J. Martín-Aparicio E. Yamamoto A. Ramos J. A.. (2002). Loss of mRNA levels, binding and activation of GTP-binding proteins for cannabinoid CB1 receptors in the basal ganglia of a transgenic model of Huntington’s disease. Brain Res. 929, 236–242. 10.1016/s0006-8993(01)03403-5 11864629

[B46] Lee C. Y. Cantle J. P. Yang X. W. (2013). Genetic manipulations of mutant huntingtin in mice: new insights into Huntington’s disease pathogenesis. FEBS J. 280, 4382–4394. 10.1111/febs.12418 23829302 PMC3770892

[B47] Liu L. Ito W. Morozov A. (2017). GABAb receptor mediates opposing adaptations of GABA release from two types of prefrontal interneurons after observational fear. Neuropsychopharmacology 42, 1272–1283. 10.1038/npp.2016.273 27924875 PMC5437887

[B48] Logie C. Bagetta V. Bracci E. (2013). Presynaptic control of corticostriatal synapses by endogenous GABA. J. Neurosci. 33, 15425–15431. 10.1523/JNEUROSCI.2304-13.2013 24068811 PMC3782622

[B75] MacDonald M. E. Ambrose C. M. Duyao M. P. Myers R. H. Lin C. Srinidhi L.. (1993). A novel gene containing a trinucleotide repeat that is expanded and unstable on Huntington’s disease chromosomes. Cell 72, 971–983. 10.1016/0092-8674(93)90585-e 8458085

[B49] Mangiarini L. Sathasivam K. Seller M. Cozens B. Harper A. Hetherington C. (1996). Exon 1 of the HD gene with an expanded CAG repeat is sufficient to cause a progressive neurological phenotype in transgenic mice. Cell 87, 493–506. 10.1016/S0092-8674(00)81369-0 8898202

[B50] Mastro K. J. Bouchard R. S. Holt H. A. Gittis A. H. (2014). Transgenic mouse lines subdivide external segment of the globus pallidus (GPe) neurons and reveal distinct GPe output pathways. J. Neurosci. 34, 2087–2099. 10.1523/JNEUROSCI.4646-13.2014 24501350 PMC3913864

[B51] McCaw E. A. Hu H. Gomez G. T. Hebb A. L. Kelly M. E. Denovan-Wright E. M. (2004). Structure, expression and regulation of the cannabinoid receptor gene (CB1) in Huntington’s disease transgenic mice. Eur. J. Biochem. 271, 4909–4920. 10.1111/j.1432-1033.2004.04460.x 15606779

[B52] Melzer S. Gil M. Koser D. E. Michael M. Huang K. W. Monyer H. (2017). Distinct corticostriatal GABAergic neurons modulate striatal output neurons and motor activity. Cell Rep. 19, 1045–1055. 10.1016/j.celrep.2017.04.024 28467898 PMC5437725

[B53] Menalled L. B. Kudwa A. E. Miller S. Fitzpatrick J. Watson-Johnson J. Keating N.. (2012). Comprehensive behavioral and molecular characterization of a new knock-in mouse model of Huntington’s disease: zQ175. PLoS One 7:e49838. 10.1371/journal.pone.0049838 23284626 PMC3527464

[B54] Nisenbaum E. S. Berger T. W. Grace A. A. (1993). Depression of glutamatergic and GABAergic synaptic responses in striatal spiny neurons by stimulation of presynaptic GABA_B_ receptors. Synapse 14, 221–242. 10.1002/syn.890140306 8105549

[B55] Okada M. Onodera K. Van Renterghem C. Sieghart W. Takahashi T. (2000). Functional correlation of GABA_A_ receptor α subunits expression with the properties of IPSCs in the developing thalamus. J. Neurosci. 20, 2202–2208. 10.1523/JNEUROSCI.20-06-02202.2000 10704495 PMC6772493

[B56] Olsen R. W. Sieghart W. (2009). GABA_A_ receptors: subtypes provide diversity of function and pharmacology. Neuropharmacology 56, 141–148. 10.1016/j.neuropharm.2008.07.045 18760291 PMC3525320

[B57] Overstreet L. S. Westbrook G. L. (2003). Synapse density regulates independence at unitary inhibitory synapses. J. Neurosci. 23, 2618–2626. 10.1523/JNEUROSCI.23-07-02618.2003 12684447 PMC6742076

[B58] Paldino E. Cardinale A. D’Angelo V. Sauve I. Giampà C. Fusco F. R. (2017). Selective sparing of striatal interneurons after poly (ADP-Ribose) polymerase 1 inhibition in the R6/2 mouse model of Huntington’s disease. Front. Neuroanat. 11:61. 10.3389/fnana.2017.00061 28824383 PMC5539174

[B59] Parievsky A. Moore C. Kamdjou T. Cepeda C. Meshul C. K. Levine M. S. (2017). Differential electrophysiological and morphological alterations of thalamostriatal and corticostriatal projections in the R6/2 mouse model of Huntington’s disease. Neurobiol. Dis. 108, 29–44. 10.1016/j.nbd.2017.07.020 28757327 PMC5675804

[B60] Rajput P. S. Kharmate G. Norman M. Liu S. H. Sastry B. R. Brunicardi C. F.. (2011). Somatostatin receptor 1 and 5 double knockout mice mimic neurochemical changes of Huntington’s disease transgenic mice. PLoS One 6:e24467. 10.1371/journal.pone.0024467 21912697 PMC3166321

[B61] Reiner A. Deng Y. P. (2018). Disrupted striatal neuron inputs and outputs in Huntington’s disease. CNS Neurosci. Ther. 24, 250–280. 10.1111/cns.12844 29582587 PMC5875736

[B62] Reiner A. Shelby E. Wang H. Demarch Z. Deng Y. Guley N. H.. (2013). Striatal parvalbuminergic neurons are lost in Huntington’s disease: implications for dystonia. Mov. Disord. 28, 1691–1699. 10.1002/mds.25624 24014043 PMC3812318

[B63] Rock C. Zurita H. Wilson C. Apicella A. J. (2016). An inhibitory corticostriatal pathway. Elife 5:e15890. 10.7554/eLife.15890 27159237 PMC4905740

[B64] Simmons D. A. Belichenko N. P. Yang T. Condon C. Monbureau M. Shamloo M.. (2013). A small molecule TrkB ligand reduces motor impairment and neuropathology in R6/2 and BACHD mouse models of Huntington’s disease. J. Neurosci. 33, 18712–18727. 10.1523/JNEUROSCI.1310-13.2013 24285878 PMC3841443

[B65] Sloviter R. S. Ali-Akbarian L. Elliott R. C. Bowery B. J. Bowery N. G. (1999). Localization of GABA_B_ (R1) receptors in the rat hippocampus by immunocytochemistry and high resolution autoradiography, with specific reference to its localization in identified hippocampal interneuron subpopulations. Neuropharmacology 38, 1707–1721. 10.1016/s0028-3908(99)00132-x 10587087

[B66] Smith G. A. Rocha E. M. McLean J. R. Hayes M. A. Izen S. C. Isacson O.. (2014). Progressive axonal transport and synaptic protein changes correlate with behavioral and neuropathological abnormalities in the heterozygous Q175 KI mouse model of Huntington’s disease. Hum. Mol. Genet. 23, 4510–4527. 10.1093/hmg/ddu166 24728190

[B67] Snowden J. S. (2017). The neuropsychology of Huntington’s disease. Arch. Clin. Neuropsychol. 32, 876–887. 10.1093/arclin/acx086 28961886

[B68] Straub C. Saulnier J. L. Begue A. Feng D. D. Huang K. W. Sabatini B. L. (2016). Principles of synaptic organization of GABAergic interneurons in the striatum. Neuron 92, 84–92. 10.1016/j.neuron.2016.09.007 27710792 PMC5074692

[B69] Szabo B. Dörner L. Pfreundtner C. Nörenberg W. Starke K. (1998). Inhibition of GABAergic inhibitory postsynaptic currents by cannabinoids in rat corpus striatum. Neuroscience 85, 395–403. 10.1016/s0306-4522(97)00597-6 9622239

[B70] Szydlowski S. N. Pollak Dorocic I. Planert H. Carlén M. Meletis K. Silberberg G. (2013). Target selectivity of feedforward inhibition by striatal fast-spiking interneurons. J. Neurosci. 33, 1678–1683. 10.1523/JNEUROSCI.3572-12.2013 23345240 PMC6618742

[B71] Tanimura A. Lim S. A. Aceves Buendia J. J. Goldberg J. A. Surmeier D. J. (2016). Cholinergic interneurons amplify corticostriatal synaptic responses in the Q175 model of Huntington’s disease. Front. Syst. Neurosci. 10:102. 10.3389/fnsys.2016.00102 28018188 PMC5159611

[B72] Tepper J. M. Koós T. Wilson C. J. (2004). GABAergic microcircuits in the neostriatum. Trends Neurosci. 27, 662–669. 10.1016/j.tins.2004.08.007 15474166

[B73] Tepper J. M. Koós T. Ibanez-Sandoval O. Tecuapetla F. Faust T. W. Assous M. (2018). Heterogeneity and diversity of striatal GABAergic interneurons: update 2018. Front. Neuroanat. 12:91. 10.3389/fnana.2018.00091 30467465 PMC6235948

[B74] Tepper J. M. Tecuapetla F. Koos T. Ibáñez-Sandoval O. (2010). Heterogeneity and diversity of striatal GABAergic interneurons. Front. Neuroanat. 4:150. 10.3389/fnana.2010.00150 21228905 PMC3016690

[B76] Waldvogel H. J. Kim E. H. Tippett L. J. Vonsattel J. P. Faull R. L. (2015). The neuropathology of Huntington’s disease. Curr. Top. Behav. Neurosci. 22, 33–80. 10.1007/7854_2014_354 25300927

[B77] Walker F. O. (2007). Huntington’s disease. Lancet 369, 218–228. 10.1016/S0140-6736(07)60111-1 17240289

[B78] Wu L. G. Saggau P. (1995). GABA_B_ receptor-mediated presynaptic inhibition in guinea-pig hippocampus is caused by reduction of presynaptic Ca^2+^ influx. J. Physiol. 485, 649–657. 10.1113/jphysiol.1995.sp020759 7562607 PMC1158034

