# Supplementary material for: Major Contribution of Somatostatin-Expressing Interneurons and Cannabinoid Receptors to Increased GABA Synaptic Activity in the Striatum of Huntington’s Disease Mice
Source: Front Synaptic Neurosci. 2019 May 14;11:14. doi: 10.3389/fnsyn.2019.00014 (PMC6527892; doi:10.3389/fnsyn.2019.00014)
Supplement: Supplementary file 1 [file Table_1.docx]

Supplementary Material

In addition to reporting absolute decay times of optically-evoked IPSCs and EPSCs, we also report adjusted decay times and time constants (tau). These values were assessed by first normalizing the amplitudes of optically-evoked IPSCs or EPSCs amplitudes to the maximal WT response amplitude for each group. Adjusted decay times were subsequently measured from the 90% to 10% point of the response amplitude for each of the normalized responses. Response time constants (τ, tau) were calculated from the peak amplitude to the baseline with the following single exponential decay-fitting function: $f\left( x \right)=Ae^{-t/}$, where A is Amplitude, *t* is time and τ is the time constant (tau). As shown in **Table S1**, the adjusted decay times and time constant measurements held the same genotype-dependent effects as the comparisons of the decay time constants that are reported in the manuscript.

**Table S1**. Adjusted Decay Times and Time constants (tau, τ) of optically-evoked IPSCs and EPSCs expressed in milliseconds (ms). Group means ± SEMs are reported. Comparisons for each decay time and tau were made between WT and Q175 cells at each age group. Significant differences were determined using Student’s t-tests where * p<0.05, ** p<0.01 and *** p<0.001.

|  | Q175 x PV-Cre | Q175 x SOM-Cre | Q175 x D1-Cre | Q175 EPSCs |
| --- | --- | --- | --- | --- |
| 2 month |  |  |  |  |
| WT | 80.7±7.2  τ = 44.7±3.7 | 61.2±4.4  τ =39.1±2.6 | n/a | 13.7±1.1  τ =9.0±0.6 |
| Q175 | 81.6±6.8  τ = 44.8±4.6 | 63.2±3.7  τ =36.3±2.2 | n/a | 14.2±1.2  τ =9.3±0.7 |
|  |  |  |  |  |
| 12 month |  |  |  |  |
| WT | 76.1±6.2  τ = 53.0±3.2 | 93.5±9.8  τ = 70.8±8.2 | 283.6±32.1  τ =208.3±30.7 | 14.4±0.9  τ =10.4±0.6 |
| Q175 | 56.3±5.7*  τ = 41.1±4.0* | 50.73±3.92***  τ = 41.8±4.0** | 264.5±60.0  τ =185.9±39.9 | 14.8±1.9  τ =10.8±1.0 |
